# A customized affordable multiplexed immunofluorescence method visualizes early changes in the mouse brain microenvironment upon laser cytoreduction

**DOI:** 10.3389/fncel.2025.1553058

**Published:** 2025-09-08

**Authors:** Santhosh Shanmugam Anandhan, Jeremy Spence, Farhana Begum, Nimrat Kaur, Dana Henderson, Sabine Hombach-Klonisch, Thomas Klonisch

**Affiliations:** 1 Department of Human Anatomy and Cell Science, Rady Faculty of Health Sciences, Max Rady College of Medicine, University of Manitoba, Winnipeg, MB, Canada; 2 Department of Pathology, Rady Faculty of Health Sciences, Max Rady College of Medicine, University of Manitoba, Winnipeg, MB, Canada; 3 Children's Hospital Research Institute of Manitoba (CHRIM), Winnipeg, MB, Canada; 4 Department of Medical Microbiology and Infectious Diseases, Rady Faculty of Health Sciences, Max Rady College of Medicine, University of Manitoba, Winnipeg, MB, Canada; 5 CancerCare Manitoba, Winnipeg, MB, Canada

**Keywords:** multiplex immunofluorescence, laser interstitial thermal therapy (LITT), laser cytoreduction, stereotactic laser ablation, mouse brain tumor

## Abstract

**Introduction:**

Multiplex immunofluorescence (mIF) utilizes distinct fluorophore-conjugated antibodies to enable the simultaneous visualization and quantification of multiple protein targets within a single tissue section. mIF allows high-resolution spatial mapping of cellular phenotypes within the native tissue microenvironment (TME). mIF facilitates the comprehensive analysis of complex biological systems, such as brain tumors, immune cell infiltration, and tissue heterogeneity. Laser interstitial thermal therapy (LITT) is a minimally invasive, hyperthermia-based laser cytoreductive method for the treatment of surgically inaccessible brain tumors, treatment-resistant epilepsy, and radiation necrosis. Laser-induced heat causes tissue damage, vascular leakage, and the appearance of heat-induced neo-antigens. There is an urgent clinical need to understand the elusive immunomodulatory roles of LITT in the brain TME. We describe a versatile, affordable, and customizable mIF method for the spatial imaging of multiple early tissue responses in post-LITT mouse brain.

**Methods:**

We have developed a customizable and affordable mIF protocol that uses standard histological and microscopy equipment to assess TME changes in formalin-fixed paraffin-embedded (FFPE) mouse brain tissue sections. We combined mIF with a laser cytoreduction workflow that uses MRI to monitor laser-induced tissue damage in post-LITT normal and tumor murine brains. Multiplex IF on individual tissue sections enabled the simultaneous spatial image analysis of multiple cellular and molecular immunotargets, including resident brain cell responses and immune cell infiltration, as exemplified with a mouse brain TME on Day 10 post-LITT.

**Results:**

We combined our mIF imaging procedure with *in-vivo* targeted laser-induced hyperthermic brain tissue ablation on FFPE mouse brain sections on Day 10 post-LITT. This enabled the spatial visualization of activation states of resident brain cells and the emergence and distribution of diverse phagocytic immune cell populations at the post-LITT site.

**Conclusion:**

Multiplex IF on mouse models of laser cytoablation treatment in non-tumor and tumor brains offers a significant advancement by aiding in our understanding of repair and immune responses in post-LITT brains. Our customizable mIF protocol is cost-effective and simultaneously investigates the spatial distribution of multiple immune cell populations and the activation states of different resident brain cells in the post-LITT brain.

## 1 Introduction

Immunofluorescence (IF) facilitates the selective staining of antigens in tissues and cells by exploiting antigen–antibody interactions (Buchwalow et al., 2015; Ehrlich, 1877). The indirect IF method utilizes primary antibodies from a host species that exhibit a high degree of sensitivity and specificity for an antigen of the target species (Coons et al., 1942). This is followed by the selective binding of fluorophore-conjugated secondary antibodies raised against the host species of the primary antibody to form an antigen–antibody complex. Upon excitation at an appropriate wavelength, the fluorophore emits light of a longer wavelength when returning to its resting state which allows visualization of the antibody target. Although directly fluorophore-conjugated primary antibodies are an alternative, they are more expensive and their use for other immunodetection methods may be limited. Concurrent utilization of multiple primary antibodies requires that these primary antibodies are from different host species which may be a limiting factor regarding quality, specificity, availability, and cost (Tan et al., 2020).

There is an emerging need for multiplexed immunodetection of more than two different antigens to elucidate dynamic alterations of cellular and molecular markers and/or illustrate complex interactions occurring within the brain microenvironment. This is particularly evident in surgical thermal Stereotactic Laser Ablation (SLA), also named laser interstitial thermal therapy (LITT). Advancements in real-time magnetic resonance (MR) imaging techniques and minimally invasive operating procedures have enabled LITT as a treatment for surgically inaccessible brain tumors and patients suffering from treatment-resistant epilepsy (Kang et al., 2016; Salem et al., 2019; Schober et al., 1993; Waseem et al., 2015). Three LITT devices have been approved for clinical applications by the United States Food and Drug Administration and Health Canada (Lee et al., 2021; Sloan et al., 2013). LITT is recommended for patients with deep-seated or hard-to-access lesions, including primary brain tumors, brain metastases, and radiation necrosis less than three centimeters in size (Rahmathulla et al., 2012; Torres-Reveron et al., 2013) as well as for brain tumors located in or close to eloquent functional areas of the brain where conventional neurosurgery would be too risky (Patel and Kim, 2020). LITT has also been successfully administered to patients with therapy-resistant recurrent brain tumors and radiation necrosis. In combination with chemo-radiation therapy, LITT was shown to improve overall quality of life and progression-free survival of brain tumor patients (de Groot et al., 2022). However, the underlying mechanisms by which LITT achieves these clinical benefits are still unknown due to the lack of suitable *in-vivo* models. We have established a LITT mouse model (Spence et al., 2024) and combined this *in-vivo* approach with an efficient and affordable multiplex immunofluorescence (mIF) labeling technique as a powerful, customizable, multi-targeted immunodetection strategy to study post-LITT cellular and molecular changes.

Upon laser activation, thermal energy is released into brain tissue, and this heat dissipates into the surrounding brain regions. Thermal ablation damage produces a laser-induced central core (coagulative necrosis) of tissue destruction, followed by a thermal gradient cone with zones of decreasing tissue damage (Lerner et al., 2022). Sections of paraffin-embedded formalin-fixed (FFPE) LITT-treated cerebral tissues should be utilized most effectively to enable and expedite studies on dynamic cellular and molecular compositional changes in serial sections, thereby reducing the number of mice required for each experiment. Spatial mIF facilitates the simultaneous examination of multiple biomarkers in the same tissue section and enables visualization of the topographic distribution of these biomarkers. Recently, mIF has undergone a remarkable development in the immune-oncology field (Andreou et al., 2022). Numerous sophisticated instruments and microscopes have been engineered to study up to 100 markers concurrently. However, the results obtained with extensively multiplexed staining procedures are often difficult to interpret. These multiplexed immunodetection assays frequently utilize primary antibodies that have not been optimized for the particular tissue under investigation. Moreover, the substantial costs of acquiring a spectral microscope and purchasing expensive assays are prohibitive for many laboratories with limited financial resources. Here, we describe an optimized protocol for a customizable and affordable mIF protocol applied to routine 5-um thick FFPE tissue sections of murine normal and allografted tumor brains treated with or without LITT. This mIF procedure is ideally suited for studying spatiotemporal cellular changes post-LITT; this includes resident brain cells and immune cells. We show mIF using seven different antibodies that have been previously optimized individually for use in the mouse brain. Our optimized mIF protocol uses a commercially available stripping reagent to remove antibodies from the section after imaging and before repeated staining and imaging with a different set of primary and secondary antibodies. Computational processing enables overlays and alignments of individual IF images to generate a multiplexed tissue image of high quality. Employing fluorophores with minimal spectral overlap, our mIF procedure utilizes a regular immunofluorescence microscopic setup available in many imaging units. Notably, the brain is among those tissues with high autofluorescence (AF) in the green (≈488 nm) spectrum, which is aggravated further by tissue damage caused by the LITT procedure which severely limits the use of this wavelength in the mIF. We describe the use of an AF quencher that significantly mitigates or completely removes autofluorescence in LITT brain tissue. Our mIF protocol of serial tissue sections at different time points post-LITT utilizes a broad spectrum of commercially available fluorescence markers and enables a comprehensive analysis of spatiotemporal immunoreactive cellular and molecular dynamics in normal brain and glioma brain tumor tissues.

## 2 Materials and methods

### 2.1 Brief description of the mouse LITT protocol

Brain tissues for mIF were obtained from post-LITT mouse brains that had been allografted with the CT2A mouse glioma cell model. Animal ethics protocols pertaining to brain allografting of murine glioma cells and LITT were approved by the Animal Care Committee at the University of Manitoba in accordance with the ethical guidelines set by the Canadian Council for Animal Care (CCAC), protocol AC11748. C57BL/6 mice aged 6–10 weeks were used as an immunocompetent model. Here, we briefly describe the individual steps of the LITT procedure. For more details, we refer to our detailed description of the LITT protocol (Spence et al., 2024).

- The surgical area and stereotactic frame (e.g., KOPF Model 940 Small Animal Stereotaxic Instrument with Digital Display Console; KOPF Instruments, Ca, USA) are set up, surgical instruments are sterile, and additional supplies required are readily accessible before the start of the surgery.

- A KOPF Model 1772-F Universal Holder with Tuohy needle support for the laser fiber and thermocouple probe was used in combination with a Needle Support Foot with two holes (KOPF Instruments, CA, USA) to keep the laser fiber and thermocouple probe separated during stereotactic brain surgery (Supplementary Figures 1a,b).
- Prior to LITT, mice had been orthotopically allografted with CT2A mouse glioma cells. Brain tumors were confirmed by magnetic resonance imaging (MRI).
- Anesthetize the animal, record the weight, shave the surgical area, and administer any supplementary fluids or pain medications.

- We recommend isoflurane anesthesia throughout the procedure rather than injectable options, as the former is continuously adjustable and surgery times may vary between animals.
- Transfer the animal to the stereotactic frame and fix the skull in a neutral orientation using the bite bar, nosecone, and ear pins. Prior to surgery, check for hind-limb reflexes to ensure adequate depth of anesthesia and apply ophthalmic ointment.
- Apply surgical scrub solutions using cotton-tipped swabs to disinfect the incision area. Three alternating 70% ethanol and chlorhexidine (or iodine-based) scrubs are recommended. Allow the final scrub to dry completely prior to making an incision.
- Use a #15 blade scalpel to make a small rostral to caudal midline incision and use scissors to lengthen the incision if necessary. The incision should be ≈5 mm caudal to the eyes to ensure blinking is not impeded after wound closure.
- Use a sterile cotton swab to move the scalp sideways and dry the skull cap to localize Bregma. Ensure an adequate region of the skull is accessible for drilling.
- Secure the laser device in the stereotactic frame and zero the coordinates with the tip at Bregma.
- Reposition the tip of the laser device over the burr hole and ensure that the hole is wide enough to accommodate the paired laser and thermocouple device without obstruction. Use a Dremel handheld drill with a small drill bit to enlarge the burr-hole if necessary, taking care not to damage the underlying brain and meninges.
- Adjust the position over the burr-hole and lower the laser fiber to the final coordinates in the striatum (+2 mm ML, +0.5 mm AP, −2.0 mm DV).
- Set the LITT parameters and engage the laser. Laser settings of 1 W power in continuous firing mode for up to 60 s provide effective and consistent ablations, but other parameters can be chosen based on the experimental goal.
- Slowly retract the laser fiber and immediately clean the tip *gently* with a cotton-tipped swab soaked in 70% ethanol so as not to damage the tip of the laser fiber.
- Close the incision using wound clips or three to four interrupted sutures with a 5–0 monofilament with a reverse cutting needle.
- For recovery from surgery, transfer the animal to a warmed recovery cage. Once recovered, return the animal to its home cage and provide moistened chow. Monitor post-operatively twice daily for a minimum of 3 days and administer pain medications or prophylactics according to institutional guidelines.

### 2.2 Brain imaging

MR images are obtained 1 day prior to the LITT procedure to confirm tumor growth and again one-day post-LITT to verify successful targeting and extent of thermal ablation. MR images were acquired using a seven-Tesla cryogen-free superconducting magnet from MR Solutions© (Boston, MA, USA) with a 17 cm bore and equipped with a dedicated quadrature mouse head coil.

- Anesthetize the animal in an induction chamber with 2.5–3.5% isoflurane vaporized with 0.6 L/min oxygen, transfer to a warmed bed, and secure using a bite bar, nosecone, and ear pins. Monitor animal respiration and adjust anesthesia as required.
- Perform a T2-weighted coronal scan of the whole mouse brain using the following parameters: fast spin echo sequence, TR 5000 ms, TE 45 ms, echo trains 7, FOV 30 × 30 mm^2^, matrix size 250 × 256, total slices 18, slice thickness 0.3 mm, with two averages.

### 2.3 Tissue collection and fixation

- Euthanize the experimental animals, ensuring that tissues are harvested immediately.
- Grossly section the brain tissues as needed and place them into a 10% buffered formalin solution of at least 20 times tissue volume to fix overnight at room temperature (Table 1).

NOTE: Fixation using trans-cardiac perfusion can also be used and may help with issues of autofluorescence from red blood cells and/or degenerating neurons. Additionally, perfusion fixation is generally preferred when using frozen sections and when collecting brains with large tumors as they can pose challenges during tissue collection. We use a slightly modified version of a published protocol with the animal being induced and maintained under anesthesia using isoflurane rather than an injectable anesthetic drug (Table 1; Wu et al., 2021).

**Table 1:** Tools and reagents used for multiplex IF.

Name	Brand	Cat. no
Microtome – Shandon finesse ME	Fisher, Waltham, MA	77,500,102
Microtome Blade S22	Fisher, Waltham, MA	12-631P
Superfrost plus slides	Fisher, Waltham, MA	1,255,015
Xylene Histological grade	Fisher, Waltham, MA	X3P-1GAL
Ethanol	Greenfield Global	P016EAAN
Baking oven isotemp	Fisher, Waltham, MA	51,030,503
Liquid blocker super pap pen	Emsdiasum, Hatfield, PA	71,310
Normal goat serum (NGS)	Sigma, Oakville, ON	G9023
Fluoromount-G	Fisher, Waltham, MA	00–4,958-02
Phosphate Buffered Saline (PBS)	Fisher, Waltham, MA	BP399-4
Triton-X 100	Sigma, Oakville, ON	T8787
TrueVIEW Autofluorescence quencher	Vector, Newark, CA	VECTSP8400
Vectaplex antibody removal kit	Vector, Newark, CA	VECTVRK1000
Coverslips	Epredia, Kalamazoo, MI	152,455
Permount	Fisher, Waltham, MA	SP15-500
Tween 20	Sigma, Oakville, ON	P1379
Humidifying chamber		
Double distilled water		
Microwave oven		
Zeiss M2 Microscope	Zeiss, Jena, Germany	

### 2.4 Paraffin processing and embedding

- Wash the brain tissues three times for 5 min each in PBS to remove the fixative solution and place them in tissue cassettes.
- Process the tissues using a tissue processor by dehydrating the tissues in an ascending ethanol series, clearing in xylene, and infiltrating with paraffin wax.
- Following tissue processing, place the tissues in the desired orientation in metal embedding molds. Embed using double-filtered paraffin wax. Perform this step using an embedding workstation (e.g., Leica HistoCore Arcadia H heated embedding workstation).
- Cool the molds on the workstation cold plate and remove the paraffin tissue blocks from the molds.

### 2.5 Paraffin sectioning

- Trim the paraffin blocks to expose the tissue surface and cool the blocks on ice with the cut surface down for 2–3 h before sectioning.
- Using a microtome (e.g., Shandon finesse ME), section the blocks as a ribbon at 5 μm thickness. Place the section ribbon onto a water bath at room temperature to flatten the sections and remove any wrinkles. Carefully collect the floating sections on Superfrost plus slides.
- Dry the sections on a slide drying rack for several minutes and then overnight on a warming plate at 40°C.

### 2.6 Hematoxylin and eosin (H&E) staining

- H&E stain every tenth slide to locate the LITT damaged site and identify the treatment coordinates to perform IF staining.
- Deparaffinize the slides in xylene and rehydrate the sections in a descending ethanol series progressing to tap water.
- Stain the tissue sections with hematoxylin and wash the slides under running tap water to remove excess stains.
- Differentiate the stain by briefly dipping the slides in acid ethanol and washing them briefly in running water.
- Blue the hematoxylin in the nuclei by immersing the slides in lithium carbonate solution and then washing the slides under running water.
- Counterstain the sections using an eosin-phloxine solution and wash them under running water. Eosin-phloxine stains the cytoplasm and other tissue components in shades of pink.
- Dehydrate the sections in an ascending ethanol series, clear in xylene, and mount the coverslips using Permount.

### 2.7 FFPE slide preparation and deparaffination

- View the H&E-stained sections to confirm LITT damaged sites and choose the correct sections to perform mIF.
- Place the slides in a glass slide holder, wrap the holder in aluminum foil, and bake the slides in an oven at 60°C for at least 2–3 h or overnight to remove most paraffin from the tissues.
- Place the slides in fresh xylene three times for 10 min each to remove any residual paraffin from the tissue. The xylene solution is good for deparaffinizing up to 40 slides before replacing.

### 2.8 Rehydration and antigen retrieval

- Rehydrate the tissue sections for 3 min each in an ethanol gradient series (100, 95, 80, 70, 60%).
- Rinse slides briefly in ddH_2_0 and wash for 10 min in PBS with 0.1% Tween 20 (PBS-T) using a jar with a magnetic stir bar placed at the bottom. Use a magnetic stirrer for all the wash steps.
- While slides undergo washing steps, prepare the citrate buffer solution in the fume hood in a 500 mL microwave-safe plastic container.
- Solution A = 21.01 g C_6_H_8_O_7_ + H_2_0 in 1 L ddH_2_O (citric acid). Solution B = (0.1 M) 29.441 g C_6_H_5_O_7_Na_3_ X H_2_O (Sodium-citrate dibasic trihydrate) in 1 L ddH_2_O.
- Mix 9 mL of solution A with 41 mL of solution B and bring the final volume to 500 mL with ddH_2_O.
- Slides in a glass rack are placed in citrate buffer; remove the metal handle before microwaving. Microwave for 3–4 min until bubbles begin to form in the solution and transfer to a 90°C water bath for 20 min.
- Carefully transfer the hot container with slides to the fume hood to cool for 15 min. Wash three times for 5 min each in 1x PBS-T (pH 7.6).

### 2.9 Permeabilization and blocking of tissues

- Prepare a 0.1% Triton X-100 solution in dd H_2_0 and incubate slides for 7 min, then wash with PBS-T three times for 5 min each.
- Carefully remove excess buffer on the slide with a paper towel and use the Liquid blocker PAP pen (EMS #71310, Hatfield, PA) to draw margins around the sections. Do not touch the sections with the pen as this will create a hydrophobic barrier and prevent reagents from reaching the tissue section, hence, rendering the section useless for the upcoming immunolocalization!
- Block non-specific binding sites by covering each section with 100 μL of 10% normal goat serum (NGS) in PBS-T in a covered humidifying chamber for 30 min at room temperature.

- NGS blocks unspecific protein–protein interactions and prevents non-specific binding of primary and secondary antibodies to the tissue.
- Prepare a 1:50 F(ab) blocking reagent using 10% NGS/PBS-T. Remove the 10% NGS/PBS-T buffer and add the F(ab) blocking solution to block the sections for 1 h at room temperature.

- F(ab) fragments are used to block endogenous immunoglobulins within the tissues and exposed immunoglobulins in multiple labeling experiments when using primary antibodies from the same species (e.g., mouse Abs on mouse tissue).

### 2.10 Primary antibody cocktail

- Prepare a primary antibody cocktail and IgG controls in PBS-T + 10% NGS.
- Prior to the mIF experiment, individual antibodies were tested separately to confirm suitability as a tissue marker, determine background staining, and optimize antibody dilution.
- Prepare the cocktail of all three antibodies at the recommended dilutions in 10% NGS/PBS-T for each tissue section. Use isotype-specific control immunoglobulins (Ig) at the same concentration as the corresponding specific antibody.
- Add 100 μL of the primary antibody cocktail (or Ig control solution) to each section, place in a humidified chamber, and incubate overnight at 4°C (Table 2).

**Table 2:** Antibodies optimized for multiplex IF.

Name	Specifics	Dilution	Source	Cat. no	RRID
Primary antibodies for 1^st^ IF cycle					
MBP Antibody (2H9)	Mouse monoclonal	1/100	Novus, Centennial, CO	22121SS	AB_3266940
NESTIN	Chicken polyclonal	1/100	Aves Labs, Davis, CA	NES-0020	AB_2314882
IBA1	Rabbit monoclonal	1/100	Wako, Richmond, VA	019–19,741	AB_839504
Primary antibodies for 2^nd^ IF cycle					
GFAP	Chicken polyclonal	1/100	Aves Labs, Davis, CA	GFAP	AB_2313547
αSmooth Muscle Actin	Mouse monoclonal	1/100	Sigma, Oakville, ON	A2547	AB_476701
CD68 (E3O7V)	Rabbit monoclonal	1/100	CST, Danvers, MA	97,778	AB_2928056
Primary antibodies for 3^rd^ IF cycle					
F4/80 (D2S9R) XP	Rabbit monoclonal	1/100	CST, Danvers, MA	70,076	AB_2799771
Primary antibodies for 4^th^ IF cycle					
CD31	Rabbit polyclonal	1/100	Abcam, Waltham, MA	ab124432	AB_2802125
Other antibodies for the experiment					
NeuN	Rabbit polyclonal	1/350	Sigma, Oakville, ON	ABN78	AB_10807945
Alexa Fluor 568	Goat anti Chicken	1/1,000	Invitrogen, Waltham, MA	A11041	AB_2534098
Alexa Fluor 647	Goat anti Rabbit	1/1,000	Invitrogen, Waltham, MA	A21245	AB_2535813
Alexa Fluor 488	Goat anti Mouse	1/1,000	Invitrogen, Waltham, MA	A11029	AB_2534088
Goat F(ab)	Anti mouse IgG H&L	1/50	Abcam, Waltham, MA	ab6668	AB_955960
DAPI	Nuclear counterstain	1/50,000	Sigma, Oakville, ON	D9564	

### 2.11 Secondary antibody incubation

Day 2 of multiplex IF: Perform the next steps in the dark.

- Wash the sections in PBS-T three times for 10 min each with the Coplin jar containing a small magnetic bar placed on a magnetic stirrer.
- Prepare a secondary antibody cocktail in PBS-T. Add 100 μL of the secondary antibody cocktail per section and incubate for 1 h at room temperature in a humidified chamber.
- Wash the sections with PBS-T three times for 10 min each.

### 2.12 Autofluorescence quenching

- The protocol was obtained from the product datasheet and optimized.
- For each tissue section, approximately 100 μL of Vector TrueVIEW Reagent (#VECTSP8400, Vector, Newark, CA) is required.
- To prepare Vector TrueVIEW Reagent, a ratio of 1:1:1 of proprietary Reagents A, B, and C is required (i.e., 33 μL Reagent A + 33 μL Reagent B + 33 μL Reagent C). The order of mixing is important!
- Add equal volumes of Reagent A and B in a microcentrifuge tube. Mix for 10 s. Add Reagent C to the mixture (ensuring a 1:1:1 volume ratio of reagents A, B, and C) and mix again for 10 s. Vector TrueVIEW Reagent is now ready to use. Once prepared, Vector TrueVIEW Reagent is stable at room temperature for at least 2 h.
- Drain excess PBS-T buffer from the tissue section. Add Vector TrueVIEW Reagent to cover the tissue section completely (≈100 μL per section) and incubate for 25 min.
- Wash in PBS-T buffer for 5 min using a magnetic stirrer and drain excess buffer from the section.

#### 2.12.1 Autofluorescence quencher optimization

- The autofluorescence quencher must be optimized for each tissue type.
- Set up an IF experiment using serial FFPE sections from a LITT-treated mouse brain.
- Process the sections until step 2.9 and then add the Vector TrueView Autofluorescence quencher to all sections. Incubate the sections for time points from 5 to 45 min.
- Wash briefly in PBS-T for 5 min, DAPI stain, and coverslip the slides [*Refer to step 2.13*].
- View the sections in a fluorescence microscope using the filter for AF488, auto-fluorescence channel, and optimize the duration of exposure with the quencher.
- Incubation for 25 min with TrueView Autofluorescence Quencher was found to be optimal for LITT-treated mouse brains.

### 2.13 Nuclear staining (DAPI incubation)

- Prepare DAPI (1:50,000) in PBS-T, add 100 μL per section, and incubate for 5 min in a humidified chamber.
- Wash with PBS-T for 5 min and remove excess buffer around the tissue before adding the mounting medium.
- Mount with Fluoromount G and coverslip. Perform imaging as outlined below before proceeding with stripping.

### 2.14 Stripping

- The protocol was obtained from the product datasheet and optimized.
- Place slides in PBS-T for 15–30 min. Carefully remove the coverslip from the tissue section and wash thoroughly with PBS-T (pH 7.4) to remove residual mounting media.
- Add sufficient VectaPlex™ Reagent A to cover the tissue section completely (70 - 100 μL) and incubate for 15 min at room temperature.
- Wash slides briefly with PBS-T.
- Add sufficient VectaPlex™ Reagent B to cover the tissue section completely (70-100 μL) and incubate for 15 min at room temperature. Wash slides with PBS-T for 5 min

### 2.15 Second cycle of immunofluorescence staining

**Figure 1:**
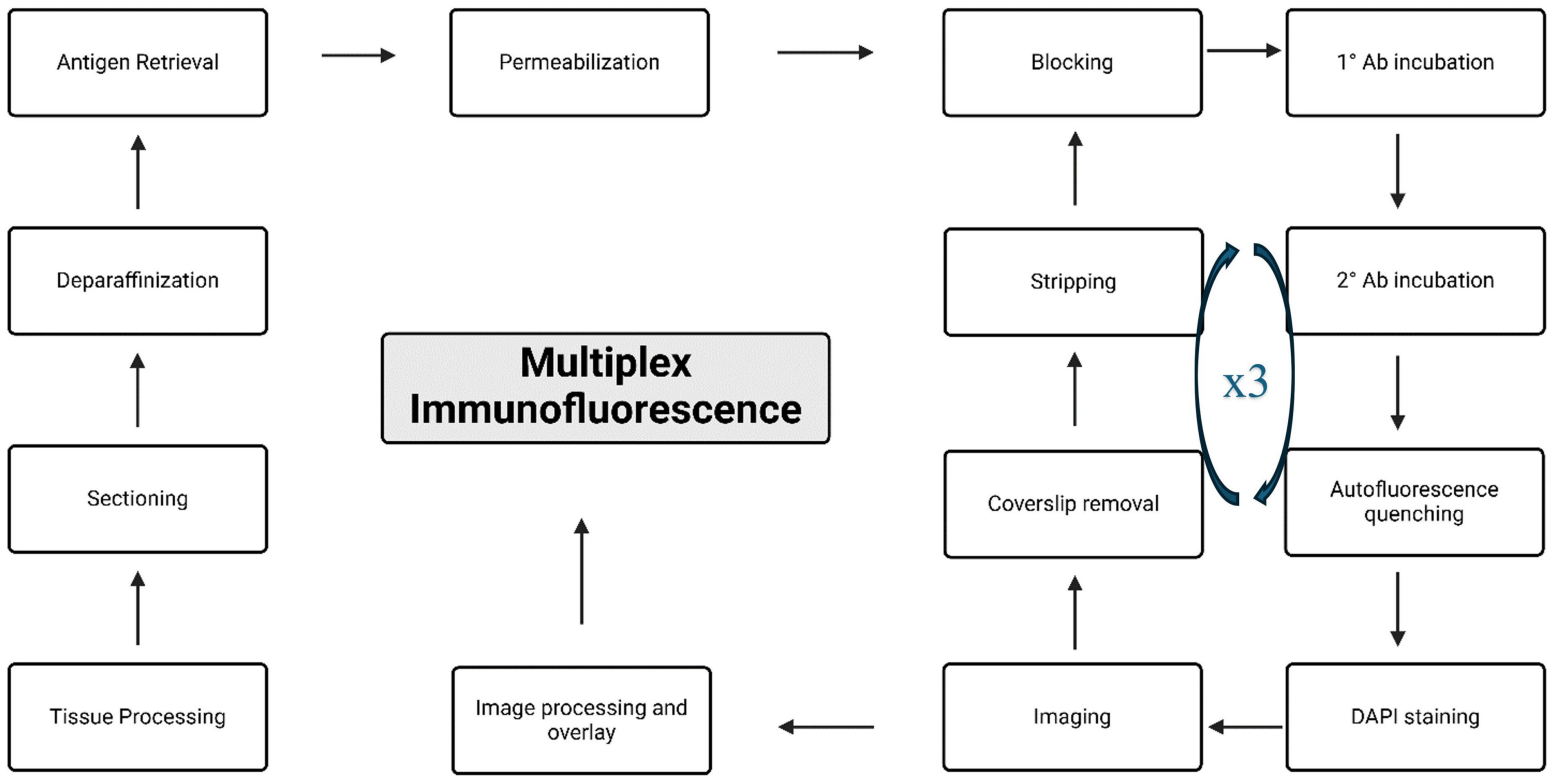
Summary of the workflow for the multiplex immunofluorescence (mIF) protocol.

- After stripping the sections, begin the second cycle of mIF by starting with the blocking step [*repeat 2.9–2.13*]. Then proceed with the primary antibody cocktail incubation overnight, followed by secondary antibody incubation, auto-fluorescence quenching, and DAPI staining on the next day.
- Imaging as outlined below, followed by the stripping step before a 3rd cycle of mIF if required.
- A schematic summary of the different steps of the mIF protocol is shown in Figure 1.

### 2.16 Tiling of images

#### 2.16.1 Image acquisition

- A Zeiss Imager M2 microscope is used with objective Zeiss 20X/0.08 ∞/0.17/OFN25, Plan-Apochromat, Zeiss Filter Set 112, and AxioCam 305 camera was used for image acquisition. Any microscope with an automated focusing and tiling option can be used.
- Open Zeiss ZEN software (ZEN 3.8 Pro), calibrate the stage, enable the tile option, and calibrate the 4-slide holder.
- Locate the region of interest and zoom in to x20 magnification.
- Mark the region to be tiled and set focus points. Multiple regions of the same slide can be marked at the same time.
- A multi-slide holder and multiple regions of interest (ROIs) can be used to calibrate and image multiple regions automatically to save time but not mandatory.
- Make sure the distributed focus points cover the entire ROI before adjusting the focus and verifying each point.
- Ensure that each region of interest is always in focus and appropriate exposure times, as determined in the single antibody optimization, are set for each marker.
- Interrupt at any time to adjust focus points. It is critically important that each tile of the image is in focus. Out-of-focus images will impair the quality of the final compiled mIF image.
- Imaging is repeated after each cycle of immunofluorescence staining.

#### 2.16.2 Image processing

- After all cycles of IF and image acquisition have been completed, open the image in the ZEN Blue.
- If you have imaged multiple tile regions within a single acquisition, the software outputs one image with multiple scenes (= ROIs).
- Use the create image subset and split option to extract the individual scenes. Under the processing tab use the stitching option to integrate the individual tiles into a single image.
- Switch channel colors to suitable pseudo-color for targeted cellular markers from different cycles of immunofluorescence. Pseudo-colors assist in distinguishing the different targeted cellular markers and aid in the visualization of their co-localization.
- Complimentary colors are used to co-localize cellular targets. Example: Red and green colors produce a yellow color when overlayed, which can identify the co-localization of two targets from different IF cycles.
- Elevate the visibility of more relevant targets by choosing a color coding with high contrast that stands out versus lighter colors for other, less relevant, markers.
- Tile-stitched .czi files can be used for ZEN Connect overlay as the preferred method. Pseudo-colors can be provided using merged multiplex images.
- Alternatively, export as pseudo-colored .tiff files to overlay them using Adobe Photoshop. Pseudo-coloring and .tiff file export are not required for overlay using ZEN Connect.

### 2.17 Imaging protocol for microscopes without tiling option

- Place the slide on the microscope, open the Zeiss ZEN software, and move to the ROI.
- Adjust the magnification and exposure time in each channel accordingly.
- Identify a distinct structure in the ROI and capture images.
- This structure in the ROI can be used as a reference to navigate the same region in successive IF cycles.
- Image subsets and stitching are not required in this case as the images are not tiled.
- Set pseudo-colors and proceed with the Image Overlay.

### 2.18 Image overlay (ZEN connect toolkit)

- The Zeiss ZEN Connect toolkit package enables the image alignment option.
- Create a new project in the ZEN Connect tab and open all the images from the different IF cycles.
- Use the first image as the reference and adjust the opacity of the second image. Hide all the other images for now. Click the second image and select the align option to hover it over the first image and precisely align. Tip: Use a unique anatomical structure from the image for alignment. Apply the alignment, hide the second image, and repeat the same with all the remaining images.
- DAPI staining is used for all the IF cycles. The DAPI signal serves as the reference channel for image merging to form a perfectly aligned single mIF image.
- Select “Custom Carrier 1” from the ZEN Connect tab to select all the images in the project and use the “Single Image Export” option to obtain a single merged image (Supplementary Figure 2a).
- In the export window, change the merge option to “Intensity-based,” export format to “CZI image multi-channel,” and pixel size to “largest”; use the default settings for the remaining options and click “Export data” to start the merging process (Supplementary Figure 2b).
- ZEN Connect merges the images tile by tile.
- The merged image will be in .czi format with all the previously assigned colors removed. The channel tabs show the details of the image along with its channel number. This can be used to identify the staining and assign the new pseudo-colors.
- Selected ROIs from the full-size merged multiplex image can be exported to .tiff files using ZEN Blue if required. Specific channel combinations (merged and individual channel images) can be selected for export to form several panels of figures. Duplicate DAPI channels can be removed.

#### 2.18.1 Image overlay using photoshop as an alternative to the ZEN connect toolkit

- To overlay images in Adobe Photoshop the originally acquired ZEN. czi images will first need to be converted to .tiff files exported with merged channel images.
- Open the .tiff files of each IF labeling cycle in Adobe Photoshop (tested using Adobe Photoshop version 25.6).
- Copy the .tiff file of the second cycle of IF and paste it onto the first cycle IF .tiff file.
- Enable the screen option to overlay only the colors from the second cycle onto the first cycle IF images.
- Like the Zeiss ZEN connect procedure, move the images over each other, match the DAPI signals, and export the .tiff file as a single mIF image.

### 2.19 Data storage

- The image file size increases with the number of channels used and the size of the tiled ROIs.
- mIF overlayed images in ZEN Connect can be compressed during image export to reduce file size and rendering time. ZEN connect “Single Image Export” function has a “JpgXr compression quality” option. Recommended compression is 75–80% (Supplementary Figure 2b).
- Zeiss ZEN Blue can be used to scale down the images while exporting the .tiff file and the images can then be used for overlay using Adobe Photoshop. We recommend scaling – 60%. Save processed .czi files of the tiled images and .tiff files.

### 2.20 Image segmentation and quantification

- We used the Intellesis software (Zeiss, Jena, Germany). Switch to the analysis tab in ZEN Blue, create a new Intellesis Trainable Segmentation model, and start training.
- Import an IF image and set “multi-channel” for training multiple targets at the same time.
- For segmentation training, use the tools in the “Labelling options” to label the positive stains for each marker and assign a class (a training categorization option) for each marker, e.g., Class 1 - DAPI, Class 2 - Iba1, and Class 3 – background (Supplementary Figure 2c).
- Add a class for background and label all the non-specific signals along with the background (include empty space in the background class).
- Once all the classes are labeled, click “train.” The segmentation results will create a new segmentation channel with all the annotations.
- Check the training preview and label more signals under each class where the software is not accurately predicting your target to improve machine learning efficiency. Train the model again and repeat this process until the Intellesis segmentation model is accurate. This trained model then needs to be exported to be used in the ZEN Blue Image Analysis workflow.
- Create a new Image Analysis program. Click Setup Image Analysis, which will open a wizard that walks you through the analysis workflow steps.
- Select “model class” and enable “fill holes” for the DAPI channel class to avoid segmentation errors. Optimize the threshold for all the classes to avoid the detection of false positive cells or cell debris.
- In the “features” step of the wizard, add “Image name,” “Count,” and other required features. Image name and count options will include the image details and number of positive cells in the analysis output.
- Preview the segmentation results and finish the training wizard to finalize and save the analysis workflow. Repeat until the model segments the images as anticipated. This analysis setting can now be run on all mIF images from your experiment to generate data tables and analysis masks for each image.

### 2.21 Quantification and statistical analysis

- Export the quantified data in Excel format.
- Measure the quantified area using the Zen Blue “rectangle” tool and normalize the total number of positive cells to the quantified area.
- The graph can be prepared using the mean number of cells representing 3 independent tissue repeats. Use GraphPad Prism version 10.2.3 software (GraphPad Prism Software, Boston, MA, USA) or similar software to prepare the graph including statistical significance.
- One-way ANOVA is used to assess the significance of 3 independent samples. Levels of significance are *p < 0.05, **p < 0.01, ***p < 0.001.

## 3 Results and discussion

### 3.1 Setting the stage for post-LITT mIF

We have previously described a standard operating procedure for LITT which consists of a pre-LITT MRI to confirm tumor growth in the mouse brain prior to LITT which is followed by a post-LITT MRI to verify the extent of LITT-induced tissue damage in the normal and tumor brain (Supplementary Figures 3a–c) (Spence et al., 2024). A comparison of pre- and post-LITT MRI images provided important quantifiable three-dimensional information on tumor location, tumor size, and extent of thermal ablation after LITT treatment and assisted with the optimization of LITT treatment parameters. MRI *in-vivo* imaging data also helped corroborate histopathology observed in H&E images collected from LITT mouse brains. The T2-weighted MRI scans identified fluid-filled spaces as hyper-intense (light shades of grey to white) areas, such as edema formation at the LITT site. Hypo-intense (black) areas within the LITT region corresponded to tissue damage and necrotic regions (Supplementary Figure 3c), as has been described in patients (Holste and Orringer, 2020; Maraka et al., 2018).

### 3.2 Considerations for improved multiplex IF results

**Figure 2:**
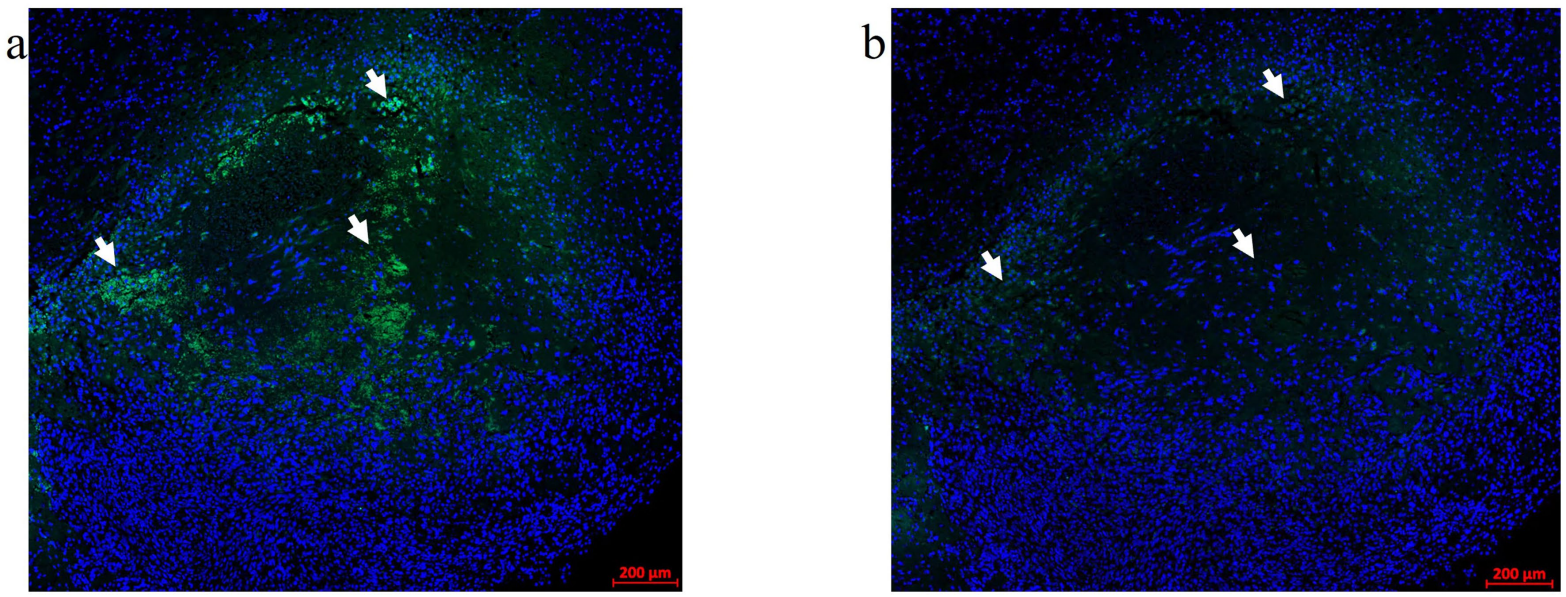
Autofluorescence quencher optimization. An FFPE CT2A mouse brain tumor-LITT section with autofluorescence (AF) in the green channel (488 nm) was used for the Vector TrueVIEW quencher reagent optimization. **(a)** Allografted CT2A tumor brain LITT region showing green tissue autofluorescence without the AF quencher. Arrows point to the autofluorescence signals. **(b)** A serial section from the same glioma tissue was treated with a Vector TrueVIEW autofluorescence quencher. Incubation times ranging from 5–25 min were tested. 20–25 min of quenching was found to be the optimal time for the AF quencher. Arrows point to the same regions as in **(a)** to indicate quenched autofluorescence.

Optimizing the incubation time for Vector TrueView autofluorescence (AF) quencher treatment ahead of immunofluorescence labeling (*see 2.12.1*) was critical in producing high-quality mIF images without autofluorescence artifacts in the green channel. The Alexa Fluor 488 nm (green) channel showed significant autofluorescence generated by blood cells and LITT-induced coagulation of proteins in and around the ablation zone. Figure 2a shows a LITT-treated mouse brain tumor tissue without the AF quencher. The incubation time with AF quencher was increased to 25 min from an initial 5 min incubation time as suggested by the manufacturer. This resulted in the complete removal of red blood cell autofluorescence without impeding subsequent immunofluorescence procedures (Figure 2b).

**Figure 3:**
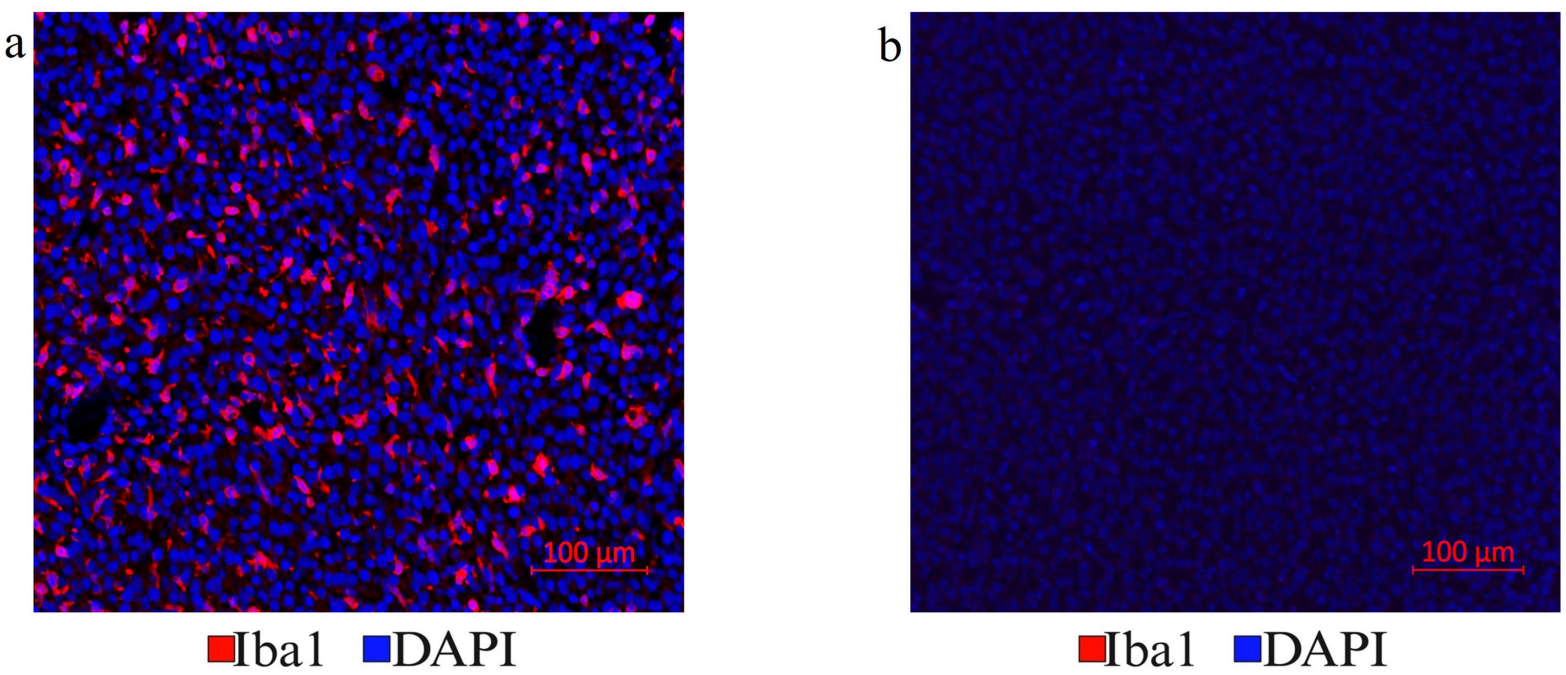
**(a)** Antibody stripping solution optimization. Mouse tumor brain tissue section with Iba1 immunofluorescence staining was chosen to optimize the Vectaplex antibody stripping solution. After imaging the Iba1 immunostaining using a goat anti-rabbit (GAR)-AF647nm antibody shown in **(a)**, the section was treated with the Vectaplex antibody stripping reagents A and B following the manufacturer’s protocol. The suggested incubation time of 15 min for each solution was used and the section was incubated again with the GAR-AF647nm antibody to check if the stripping solution had removed the primary antibody Iba1 along with its secondary antibody GAR-AF647nm. **(b)** Shows the same tissue section as in **(a)** but after antibody stripping.

The optimization of the treatment with *Vectaplex antibody stripping solution* is critical. Incomplete stripping of antibodies from a previous immunostaining step can cause aberrant tissue immunofluorescence. To accomplish optimal antibody stripping, we used an antibody to microglial marker Iba1 followed by an Alexa Fluor 647 nm labeled goat anti-rabbit (GAR) secondary antibody for visualization and imaging of immunoreactive Iba1 sites on FFPE brain tumor tissues (Figure 3a). Following the imaging of the slides, coverslips were removed, and the tissue sections were incubated with a stripping solution. Stripped tissue sections were again blocked with goat serum and incubated exclusively with the Alexa Fluor 647 nm labeled GAR secondary antibody and DAPI to stain nuclei. No positive signal was detected, demonstrating that the stripping solution had completely removed any tissue-bound rabbit primary antibody to Iba1 (Figure 3b). Tissue histology and DAPI staining remained unaffected as noted during image overlay, indicating that stripping did not result in tissue damage or a general loss of fluorescence signals.

A third cause for aberrant fluorescence signals during mIF is fluorescence bleed-through caused by overlapping excitation and emission spectra of bound fluorophores. Although the above mIF protocol can accommodate antibodies with weaker antigen binding affinity after the 3rd or 4th cycle of stripping, it is advisable to employ antibodies requiring long exposure times in the first two cycles of the mIF protocol. Antibodies with longer exposure times, like Iba1 and intermediate filament nestin (Nes), were detected in the first cycle of mIF along with the myelin marker Mbp (myelin basic protein). The macrophage marker CD68, astrocytic glial fibrillary acidic protein (Gfap), and αSma (alpha-smooth muscle actin) labeling vessel walls are candidates for the 2nd cycle of mIF, as they require shorter exposure times for detection (<400 ms). This purposeful sequence of antibody applications significantly reduces fluorescence bleed-through. Once these steps of controlling aberrant fluorescence signals have been optimized, a series of LITT tissue sections can generate highly informative mIF images.

### 3.3 Machine-learning assisted cell segmentation and quantification of mIF images

The purpose of mIF imaging is to generate quantifiable high-contrast and high-resolution spatial visualization of complex tissues from multiplexed image stacks overlayed using flexible antibody panels without the need for expensive multiplex-specific systems. We have achieved this goal using Zeiss microscopy and image processing software to import, merge, and crop the tiled images. Zeiss image quantification software was used to assign pseudo-colors to immunoreactive cellular and molecular determinants of particular interest for increased visibility and spatial quantification of multiplexed tissue images. Image analysis was performed using either the merged multiplex image with all the immune markers present or the images from a single IF cycle. Although the Photoshop overlay method can be used as the final output, we found that this software failed to retain the bio-format (raw image from the microscope), thus, making the analysis of individual markers difficult. Currently available popular analysis software in the histology field suitable for this task include QuPath, ImageJ, Zeiss Intellesis, and Zeiss Arivis Pro. QuPath and ImageJ are open-source software, whereas the others are subscription-based. We selected ROIs for the quantification of mIF images with the ZEN software. When quantifying smaller ROIs, these selected regions can be cropped out of the original image using ZEN processing prior to analysis. Zeiss Intellesis software utilizes machine learning algorithms that need to be trained on one or multiple images from the experiment prior to exporting the trained model to the analysis workflow to quantify all the other images from the same experimental group, including control images. The Intellesis training module separates DAPI, background, and target of interest into different classes as outlined by the user. Each class must be selected and several positive signals for each class must be marked and trained. The software learns and segments all the cells of similar intensity and morphology and assigns them to different classes according to the user-trained model. This model is then saved and used for all the samples with the same (immuno-)staining. Multiple targets of interest can be trained simultaneously if a multi-channel image is imported into Intellesis for training.

### 3.4 Parameters of a successful laser tissue ablation application

**Figure 4:**
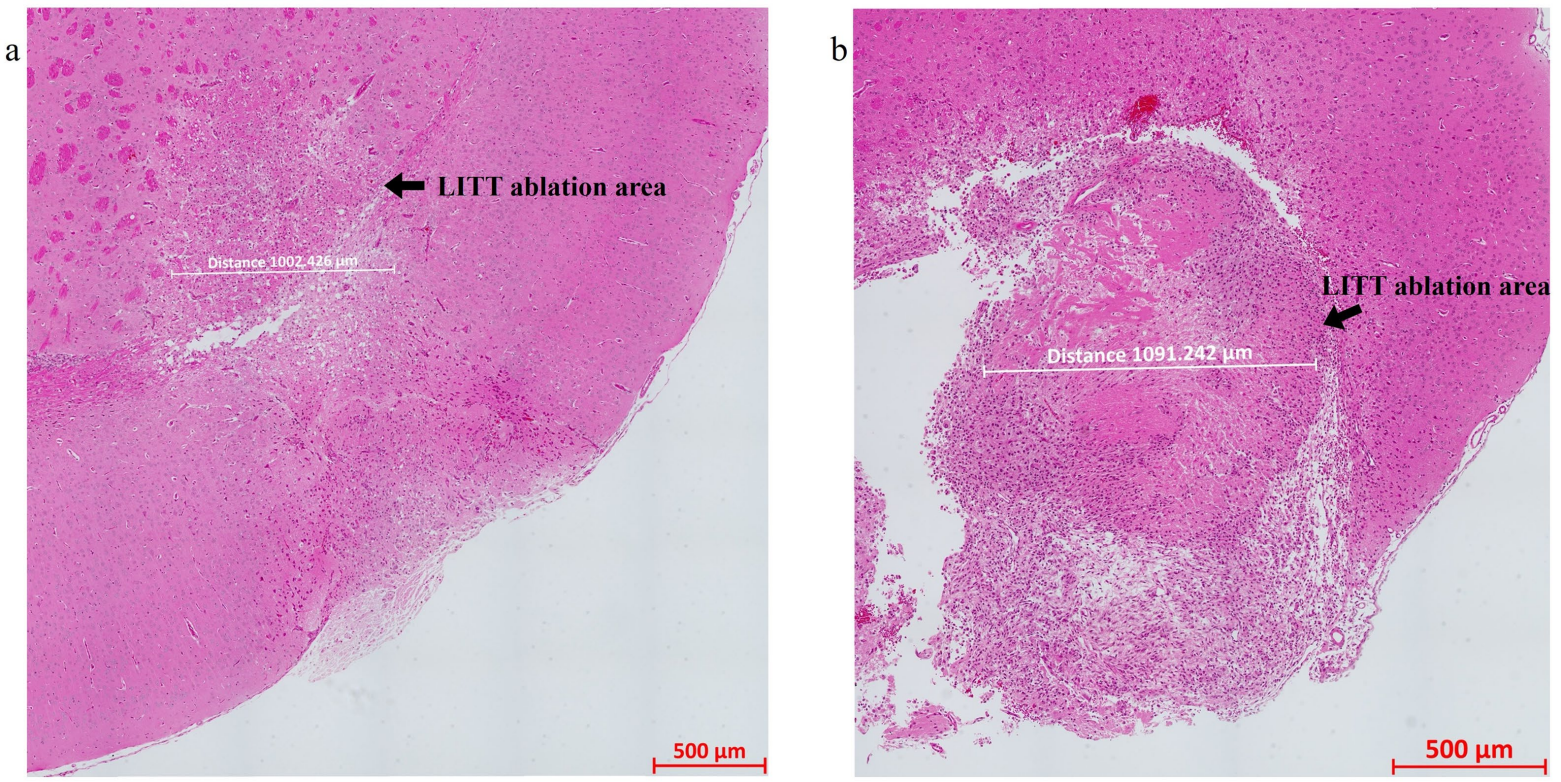
H&E-stained image of the **(a)** LITT-treated normal mouse brain and **(b)** LITT-treated tumor mouse brain. The LITT tissue damage (arrow) and the dimension of the damage are indicated. Consecutive sections of the same tissues were used for the multiplex immunofluorescence staining shown in subsequent figures.

Hematoxylin and Eosin (H&E) stained sections of LITT-treated normal brain (Figure 4a) and LITT-treated tumor brain (Figure 4b) provide histological context. Hematoxylin stains the nuclei blue/purple, whereas eosin-phloxine stains the cytoplasm, collagen, and extracellular matrix in shades of red. The degree of laser-induced tissue damage gradually decreases with increasing distance from the laser probe (Lerner et al., 2022) (Figures 4a,b). The ablation core at the site of the laser fiber tip is marked by a zone of complete tissue necrosis and eosinophilic protein coagulation with little hematoxylin stain (pyknotic and/ or destroyed nuclei) and an outer layer of cells adjacent to this ablation core. Overly aggressive LITT treatment can carbonize brain (tumor) tissue, irreparably damage the laser tip, and may result in the loss of experimental animals. Importantly, LITT treatment can cause fragility of brain tissue at the ablation site and the insertion canal, which can cause major challenges when attempting to collect the intact LITT mouse brain. We found that a slow retraction of the laser probe and thermal couple and intracardial brain perfusion fixation at the time of brain collection both aided in the recovery of intact LITT-treated brains. Minimal insertion canal damage and retention of the ablation core region within the FFPE sections are indicators of optimized LITT treatment, brain tissue processing, and sectioning protocol.

### 3.5 Spatial multiplexed IF imaging of LITT brain

**Figure 5:**
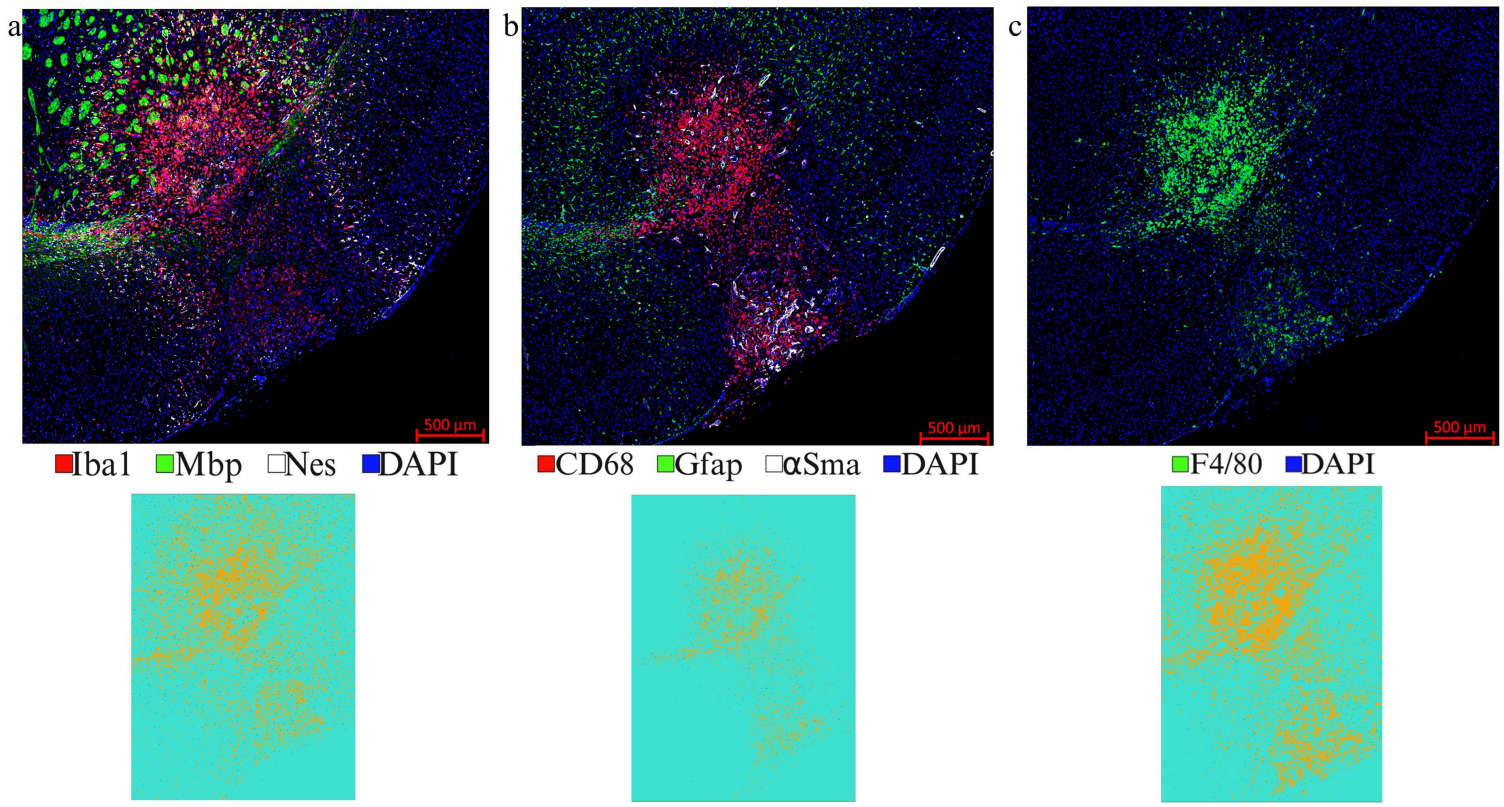
Representative images from different IF rounds. **(a)** LITT-treated normal mouse brain at Day 10 post-LITT after the 1st round of co-IF with antibodies specific for Iba1, Mbp, and Nestin is shown. **(b)** 2nd round of co-IF with antibodies for CD68, Gfap, and αSma are shown. **(c)** 3rd round of IF with F4/80 antibody is shown. Individual channel images can be extracted from different rounds of co-IF and merged and aligned with DAPI to form multiplex IF datasets for further analysis. Iba1, CD68, and F4/80 segmentation models were trained and quantified using Zeiss Zen Intellesis for non-tumor LITT mouse brains. The Intellesis trained segmentation mask of the tiled image for each of the three markers is shown below the IF image. The quantitation is shown in Figures 6f,g,h. Significance was identified using one-way ANOVA (*n* = 3). Trained segmentation models are shown for each quantified data. Constant exposure time and histogram setting were applied to all the samples.

**Figure 6:**
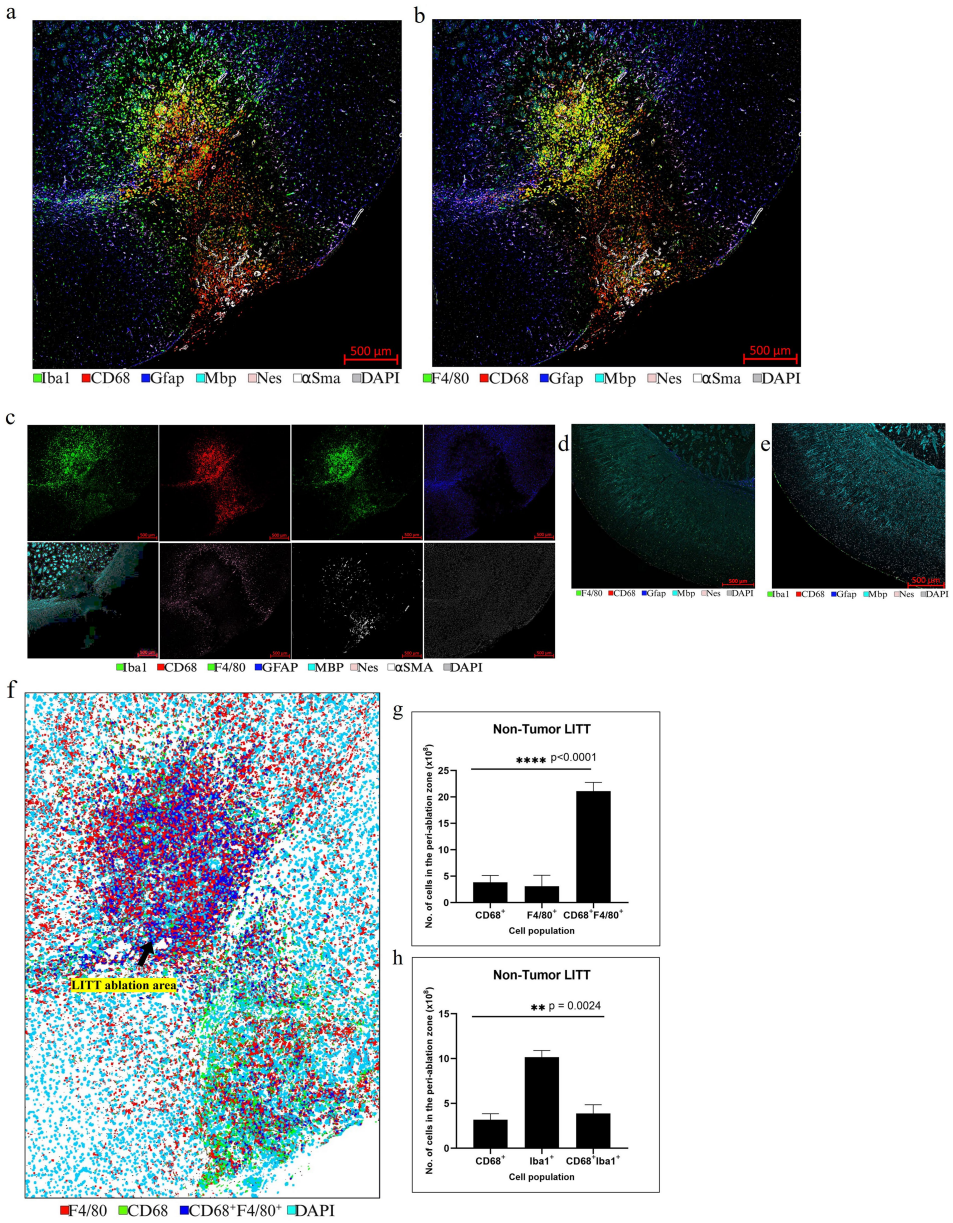
Representative selection of different markers for mIF in non-tumor brain. **(a)** mIF image of LITT-treated normal mouse brain at Day 10 post-LITT is shown for six selected markers from the first and second round of immunostaining, plus DAPI nuclear stain, as depicted in Figures 5a,b. **(b)** mIF image is shown for six selected markers from the first, second and third round of staining shown in Figures 5a–c, with CD68 exchanged for F4/80 macrophage marker. **(c)** Single-channel images for each antigen are shown. **(d,e)** Normal mouse brain control section immunostained with the five markers CD68, Gfap, Mbp, Nes, F4/80 or Iba1, and DAPI as nuclear stain reveals the presence of dense myelination (Mbp^+^). Normal mouse brain lacked Gfap^+^ reactive astrocytes, F4/80^+^ macrophage infiltration, and Iba1^+^ reactive microglia. **(f)** Representative example of a non-tumor brain segmentation mask showing cells immunoreactive for CD68 (green), F4/80 (red), and CD68, F4/80 co-immunostaining (blue). **(g)** Image quantification for the enumeration of exclusively CD68^+^ and F4/80^+^ immunopositive cells and CD68^+^, F4/80^+^ co-stained cells in normal mouse brain at Day 10 post-LITT. **(h)** Image quantification of the number of CD68^+^, Iba1^+^, and CD68^+^, Iba1^+^ cells in the normal mouse brain at Day 10 post-LITT. The total numbers of cells immunopositive for CD68^+^, F4/80^+^, Iba1^+^ and co-stained CD68^+^, Iba1^+^ and CD68^+^, F4/80^+^ cells were normalized to the total area of quantification. Significance was determined by one-way ANOVA and Tukey’s *post-hoc* test (*n* = 3).

**Figure 7:**
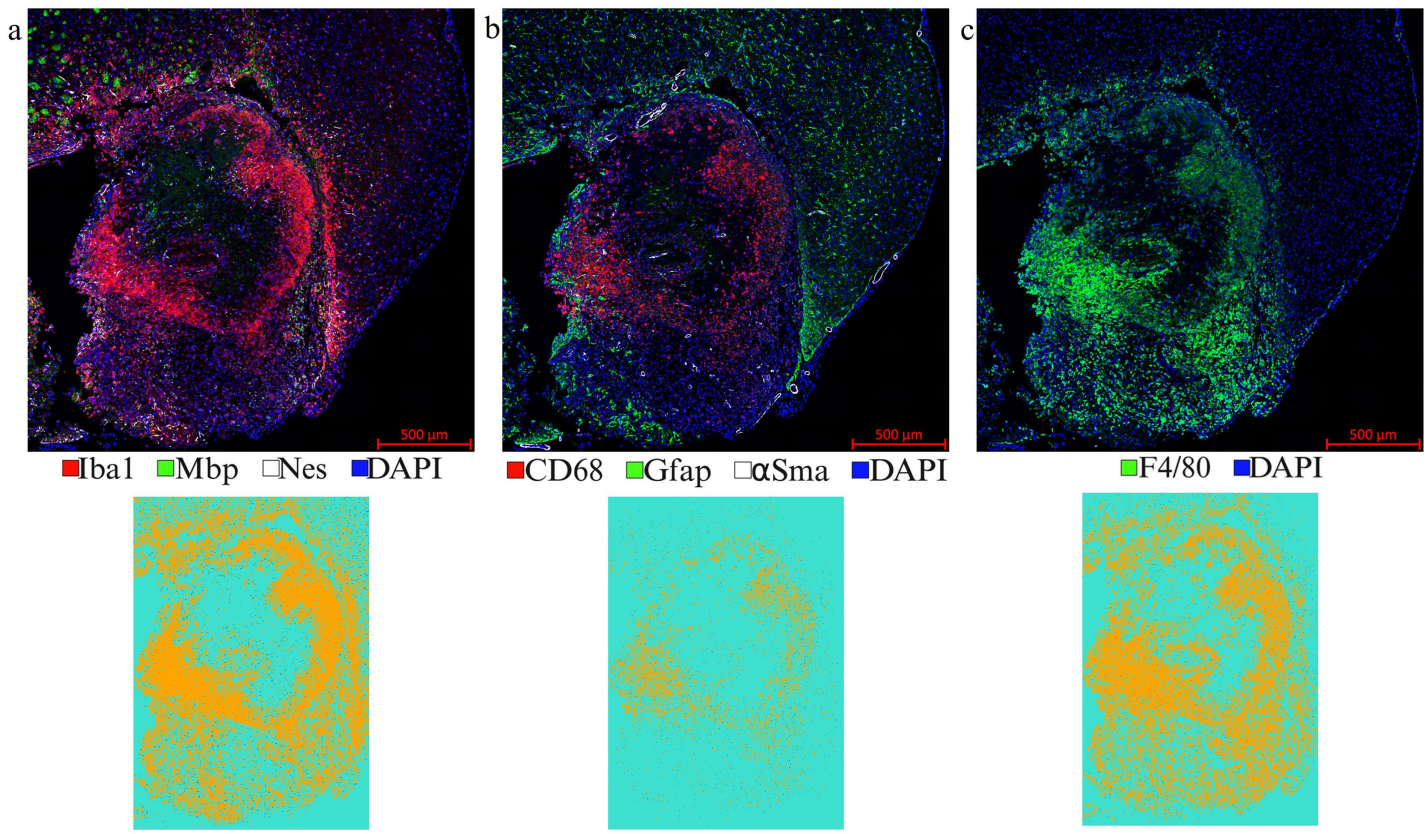
Representative images from a LITT-treated tumor brain. **(a)** 1st round of co-IF with antibodies specific to Iba1, Mbp, and Nestin; **(b)** 2nd round of co-IF with antibodies specific to CD68, Gfap, and αSma; **(c)** 3rd round of IF with F4/80 antibody were performed on a LITT-treated CT2A tumor mouse brain at 10 days post-LITT treatment. Iba1, CD68, and F4/80 segmentation models were trained and quantified using Zeiss Zen Intellesis on tumor LITT mouse brains. The quantitation if shown in Figures 9f,g,h. Significance was determined using one-way ANOVA (*n* = 3). Trained segmentation models were shown for each quantified data. Constant exposure time and histogram setting were applied to all the samples.

**Figure 8:**
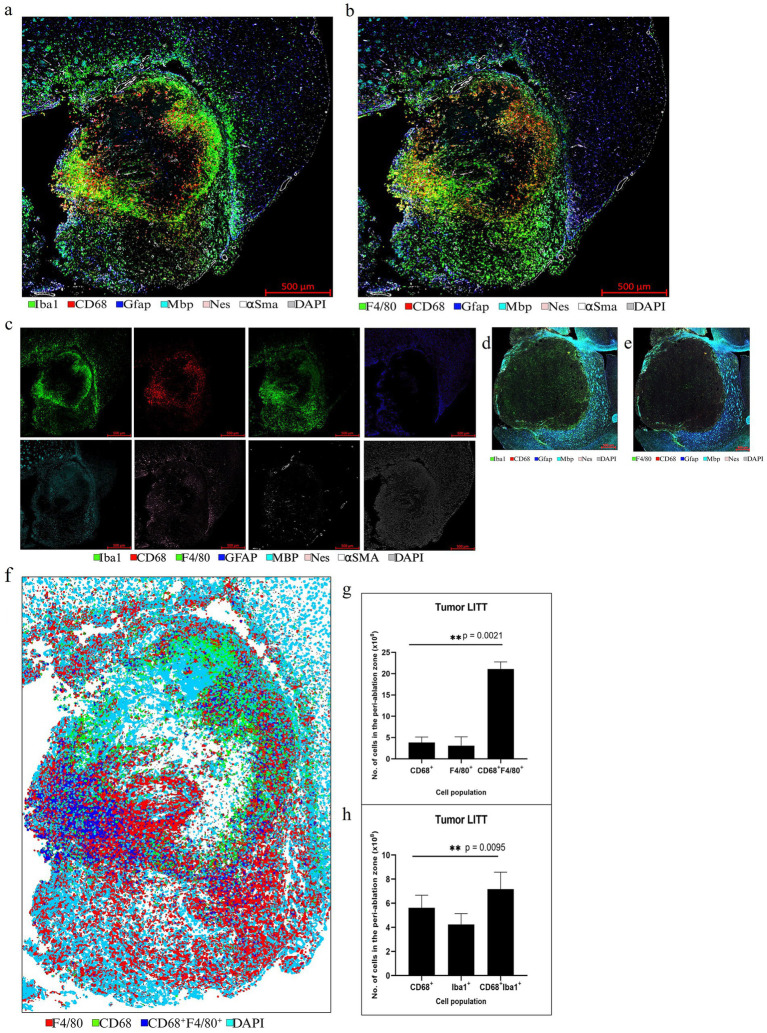
Representative selection of different markers for mIF in CT2A tumor brain. **(a,b)** mIF for innate immune cell panel performed on a LITT-treated CT2A tumor mouse brain at 10 days post-LITT treatment. **(c)** Single-channel images for each antigen are shown. **(d,e)** CT2A tumor brain with no LITT treatment as control stained with 5 different markers: CD68, Gfap, Mbp, Nes, DAPI, and F4/80, and Iba1. The images show the presence of dense myelination (Mbp^+^) outside the tumor and reactive astrocytes (Gfap^+^) in the tumor periphery. The controls lack the dense Iba1^+^ and F4/80^+^ immunostaining within the tumor as seen in the LITT tumor model. **(f)** Example of a segmentation preview of the tumor brain shows cellular immunostaining for CD68^+^ (green), Iba1 + (red), and CD68^+^, F4/80^+^ co-staining (blue). **(g)** Image quantification of the number of CD68^+^, F4/80^+^, and CD68^+^ F4/80^+^ immunopositive cells in CT2A tumor mouse brain at Day 10 post-LITT. **(h)** Image quantification of the number of CD68^+^, Iba1^+^, and CD68^+^, Iba1^+^ cells in the CT2A mouse brain at Day 10 post-LITT (*n* = 3) were immunostained and analyzed. The total numbers of immunopositive cells (CD68^+^, F4/80^+^, Iba1^+^, CD68^+^, Iba1^+^, and CD68^+^, F4/80^+^) were normalized to the total area of quantification. The significance was determined by one-way ANOVA and Tukey’s post-hoc test (*n* = 3).

**Figure 9:**
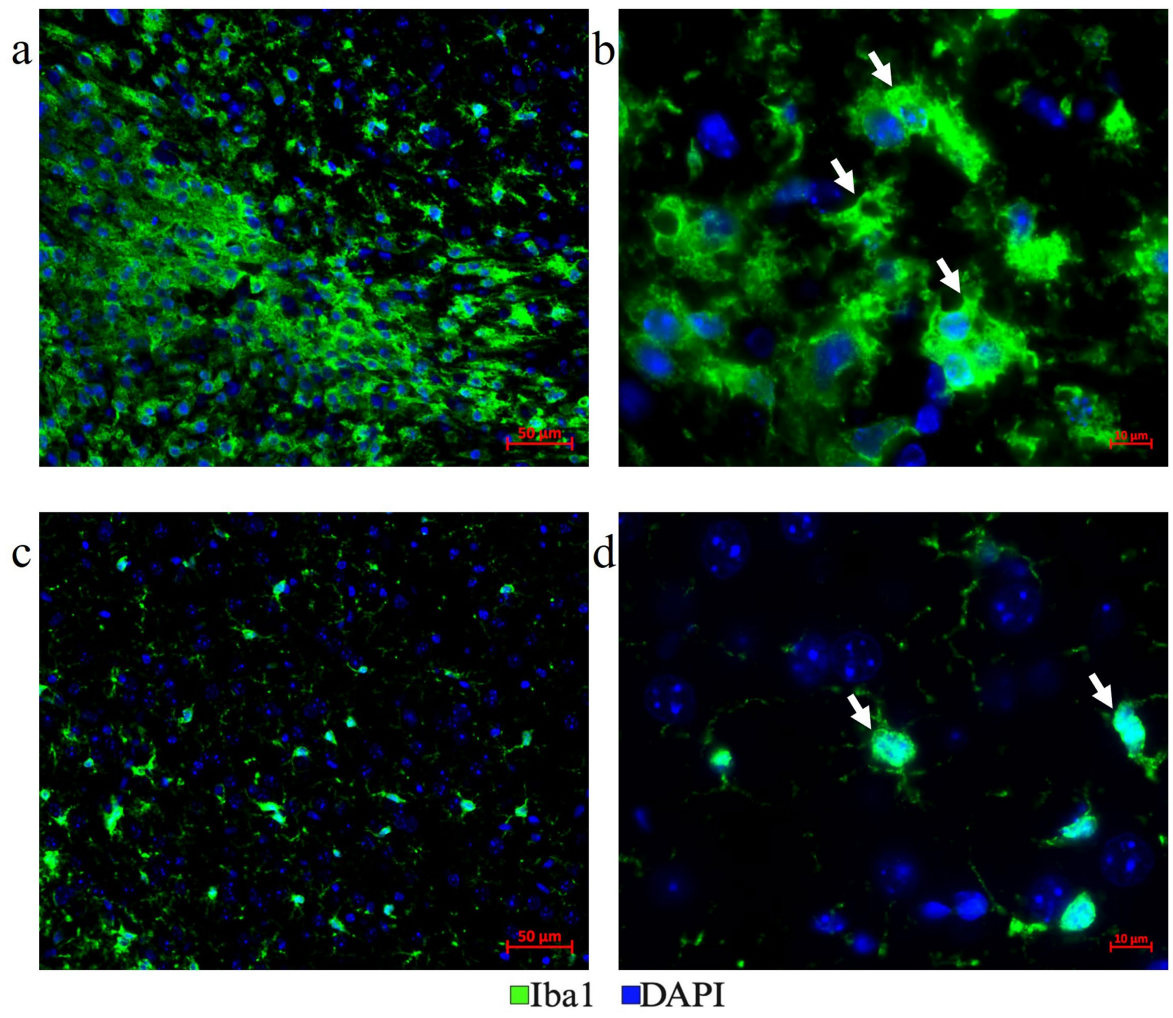
Distinct microglia phenotypes. **(a,b)** Plump amoeboid-shaped phagocytic Iba1^+^ microglial cells were located in the LITT damaged area and close to the LITT ablation core (**a-**200x mag., **b-**630x mag.). **(c,d)** Ramified reactive microglia with frequent extensions were located at a distance from the LITT damage area (**c-**200x mag., **d-**630x mag.).

LITT targets the brain tumor core to cause thermal ablation of glioma tissue. This generates cell detritus and damaged cells as a source of new antigenic sites. This can immunomodulate resident brain cells and attract innate immune cell populations from outside the brain, including monocyte- and bone-marrow-derived phagocytic cells. These cells help clear the debris field and prime later adaptive immune responses. The activation of resident brain cells and the peripheral immune cell infiltration contribute to an inflammatory brain microenvironment enriched in damage-associated molecular pattern (DAMP) signaling (Liesz et al., 2015). A major role of resident microglia is the clearance of dead cells and debris after brain injury; the latter includes the insertion of the laser fiber/ thermocouple combination and the laser-induced thermal tissue damage (Alam et al., 2020). The occurrence of Iba1^+^ resident microglia was observed early upon brain injury and the microglia is considered the first line of innate immune cell response in the LITT brain (Donat et al., 2017; Lier et al., 2020). At Day 10 post-LITT, multiple rounds of staining combinations were merged to form different panels of mouse normal brain LITT (Figures 5a–c, 6a–c) and tumor brain LITT (Figures 7a–c) mIF images. LITT-treated normal brain (Figures 5a–c, 6a,b) and tumor brain (Figures 7a–c) at Day 10 post-LITT showed activated Iba1 + microglia adjacent to the ablation core. The general macrophage marker CD68 is a surface marker detected on microglia and distinct phagocytic cell populations that have entered the LITT brain (Figures 6b,c,f,g, 7c, 8b, c, f). At the LITT ablation core, the presence of F4/80 + and CD68 + cell populations suggested the infiltration by myeloid- and monocyte-derived macrophage sub-populations into the LITT ablation core, respectively. Zeiss Intellesis and statistical analysis integrated quantification method supported the findings with an accurate spatial segmentation map of macrophage sub-populations. The quantitative analysis of the segmented mIF image revealed a significantly higher number of CD68 + macrophages co-expressing F4/80 in non-tumor (Figures 6f,g) and tumor brain (Figures 8f,g), suggesting that LITT tissue damage attracts an infiltration of non-resident macrophage populations. The activation of Iba1 + microglial cells at the focal laser injury may occur rapidly and coincides with morphological changes to Iba1 + microglial cells from a plump amoeboid shape near the LITT core (Figures 9a,b) to a ramified phenotype with frequent cell extensions was detected in the peri-ablation zone (Figures 9c,d). Iba1 + reactive microglial cells were most abundant in the peri-ablation zone together with a distinct subpopulation of Iba1 + macrophages co-expressing CD68 + in the LITT non-tumor brain (Figures 6a,c,f,h) and LITT tumor brain (Figures 7a, 8a,c,h) (Caplan et al., 2020; Umpierre et al., 2020). In mouse tumor brains treated with LITT, we observed a distinct spatial accumulation of Iba1^+^, CD68^+^, and F4/80^+^ phagocytic cell populations (Figures 7a–c, 8a,b). The single-channel images (Figure 8c) of LITT-treated and sham control tissues of CT2A tumor brains (Figures 8d,e) identified LITT as the stimulus that activated resident microglia and caused brain infiltration of CD68^+^ and F4/80^+^ macrophages; the latter were absent in normal brain sham controls (Figures 6d,e). We found Iba1^+^ microglia within viable brain tissue surrounding the LITT core, whereas CD68^+^, Iba1^neg^ phagocytic cells had a broader distribution and were detected within the debris field of the LITT core, as shown and quantified for non-tumor (Figures 6a,b,f–h) and tumor brain at Day 10 post-LITT (Figures 7a,b,f–h). This suggests that spatial differences in phagocytic cell mobility and differentiation may affect the distribution and activity of phagocytic cells at the LITT site (Figures 9a–d).

Astrocytes are the most abundant glial cells in the central nervous system and are important for maintaining blood–brain barrier integrity, nutrient support for neurons, and extracellular ion homeostasis (Escartin et al., 2021; Yuan and Wu, 2022). Along with other resident brain cells and immune cells entering the brain, astrocytes contribute to inflammation, immune responses, and tissue injury repair (Braun et al., 2017; Nespoli et al., 2024; Shinozaki et al., 2017; Wang et al., 2020). Pathological conditions, such as glioma and traumatic brain injury, cause astrocytes to change their morphology and strongly increase Gfap expression, which is indicative of elevated astrocyte activation (Lumpkins et al., 2008; Sofroniew, 2005). Gfap+ reactivate mouse astrocytes were abundantly present adjacent to LITT sites in normal brain (Figures 5b, 6a,b) and CT2A glioma tissues (Figures 7b, 8a,b). The role of reactive astrocytes in the LITT brain microenvironment requires further investigation.

**Figure 10:**
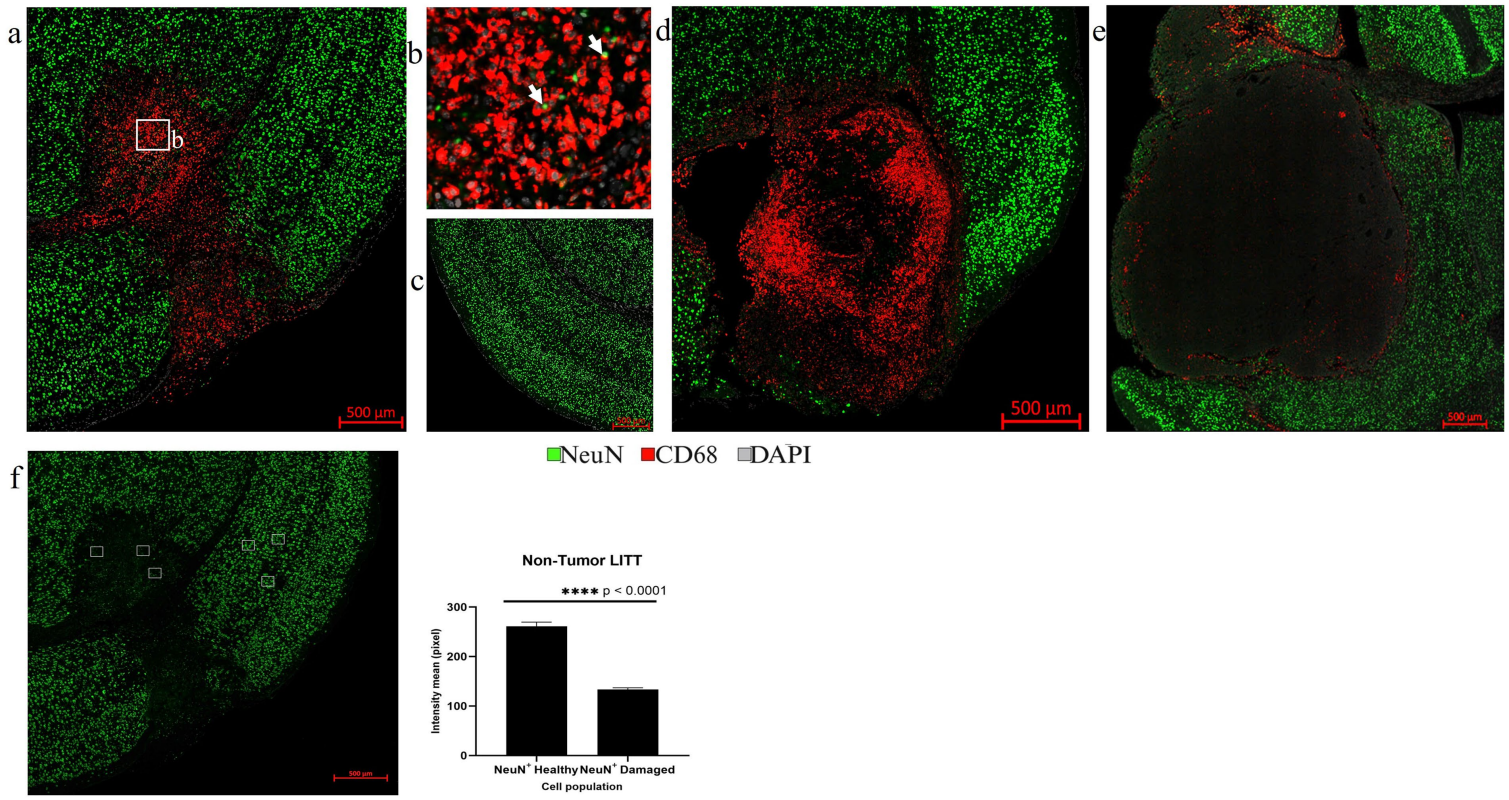
Assessment of neurons by NeuN IF. **(a)** The overview image of NeuN (green) and CD68 (red) co-stained LITT-treated non-tumor mouse brain showed the presence of CD68^+^ cells in the LITT damaged area which is devoid of NeuN immunostaining. **(b)** The zoomed-in image from **(a)** shows CD68^+^ phagocytic cells engulfing pyknotic NeuN^+^ nuclei close to the necrotic core. **(c)** Healthy control brain with no CD68^+^ cells and normal neuronal distribution. **(d)** CT2A tumor brain upon LITT treatment was stained for CD68^+^ phagocytic cells and NeuN^+^ neurons. **(e)** CT2A tumor mouse brain sham control. **(d,e)** Both show the presence of NeuN^+^ neurons outside the tumor. **(f)** Three randomly selected areas in the LITT ablation core and in cortical brain regions, respectively, were chosen for NeuN fluorescence intensity quantification in LITT-treated non-tumor brain. Intensity mean values for NeuN were calculated for NeuN^+^ neurons in the LITT ablation zone and compared with the cortical brain regions in the same mouse brain section. Significantly lower NeuN^+^ immunostaining intensity was observed in the ablation core. NeuN^+^ intensity in tumor LITT brains was not calculated because of the absence of neurons within the tumor core targeted by LITT. Hence, no damage was observed to the neurons (NeuN) and myelin (Mbp) surrounding the tumor. Significance was determined by paired t-test (*n* = 3).

Multiplex IF is well suited to assess the LITT-induced early neuronal and myelin damage in Day 10 post-LITT normal (Figures 10a–c,f) and tumor mouse brains (Figures 10d,e). Neuronal nuclei marker (NeuN) was used to differentiate neuronal nuclei from other nuclei present in cells surrounding the LITT region. A significant reduction in NeuN fluorescence intensity with the appearance of pyknotic neuronal NeuN+ nuclei surrounded by CD68 + phagocytes was typically found at the ablation core in post-LITT non-tumor brains (Figures 10a,b,f) but was absent in normal brain (Figure 10c) or tumor brain where LITT or sham effects were confined to the tumor (Figure 10d) or sham (Figure 10e) tissues.

**Figure 11:**
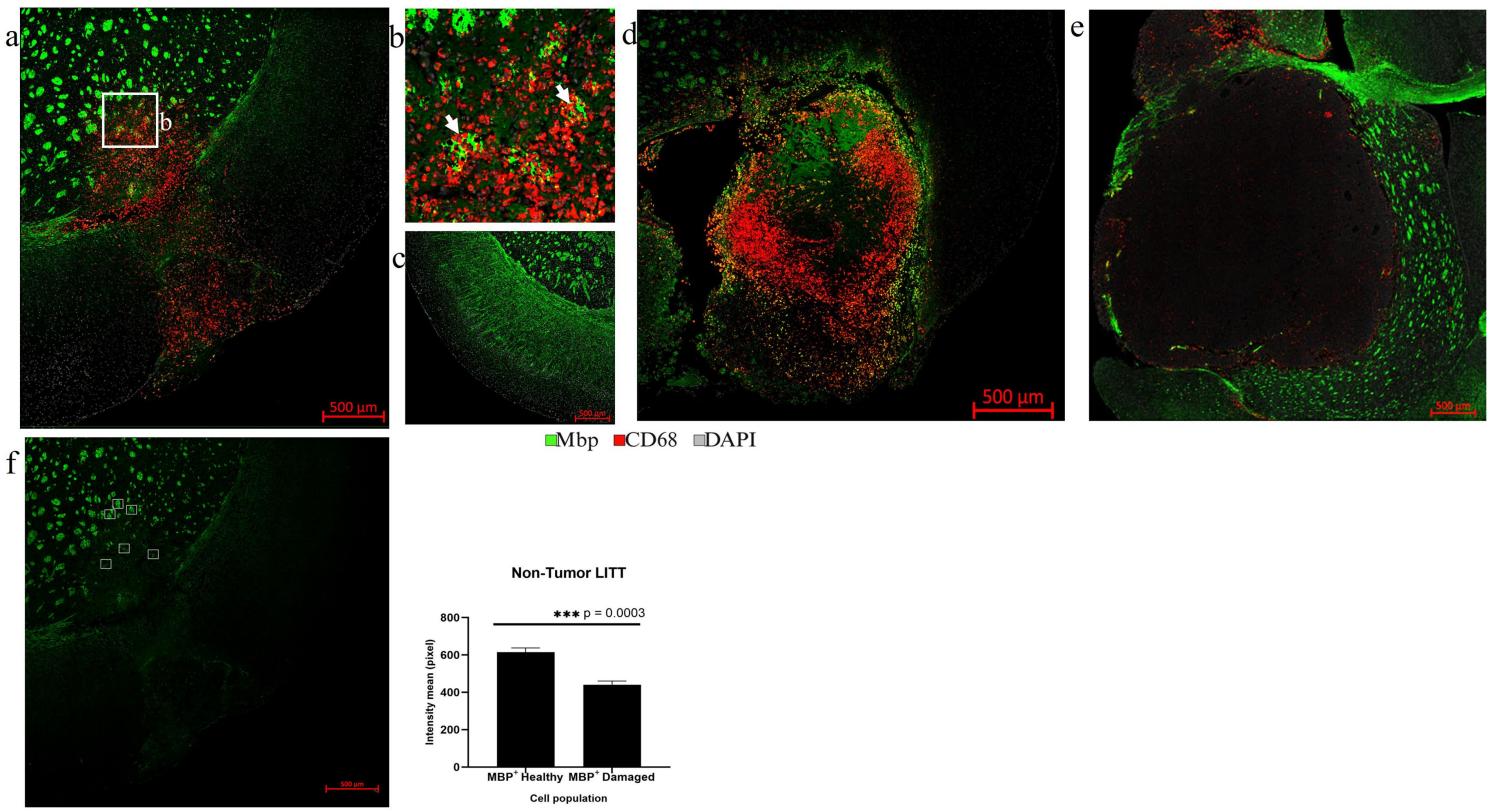
Assessment of myelin damage. **(a)** The overview images of Mbp (green) and CD68 (red) co-immunostaining in LITT-treated non-tumor mouse brain showed the presence of CD68^+^ cells in the LITT-damaged area with fragmented Mbp^+^ myelin bundles. **(b)** Zoomed-in image from **(a)** shows CD68^+^ cells near fragmented Mbp^+^ structures close to the ablation core. **(c)** Healthy control brain with intact Mbp^+^ myelin structures lacking CD68^+^ cells. **(d)** CT2A tumor brain upon LITT treatment was stained for CD68 + phagocytic cells and Mbp + myelin bundles. **(e)** CT2A tumor mouse brain sham control. **(d,e)** Both show the presence of Mbp^+^ myelin outside the tumor. **(f)** Three randomly selected areas within and outside of the LITT ablation core, respectively, were chosen for intensity based quantification of Mbp^+^ immunostaining in LITT-treated non-tumor brain. Significantly lower Mbp^+^ immunostaining intensity was observed in the selected areas of the ablation core as compared to the areas in the surrounding brain, suggesting marked damage to myelin bundles. Mbp^+^ fluorescence intensity in tumor LITT brains was not calculated since Mbp myelin immunostaining was absent in the tumor. Significance was determined by paired t-test (*n* = 3).

Myelin bundles and fibers immunoreactive for myelin basic protein (Mbp) were found fragmented in the LITT regions (Figures 11a,b). We observed significant myelin damage at the LITT site in the non-tumor LITT brains (Figures 11a,b) compared to the healthy brain control (Figure 11c). Mbp + structures co-localized with CD68 + macrophages that appeared to engulf the fragmented myelin bundles (Figures 11a,b). This coincided with a significant reduction in fluorescence intensity for Mbp at the LITT ablation site (Figure 11f). In tumor brain with LITT and sham effects confined to the glioma tissue, myelin remained unaffected (Figures 11d,e).

**Figure 12:**
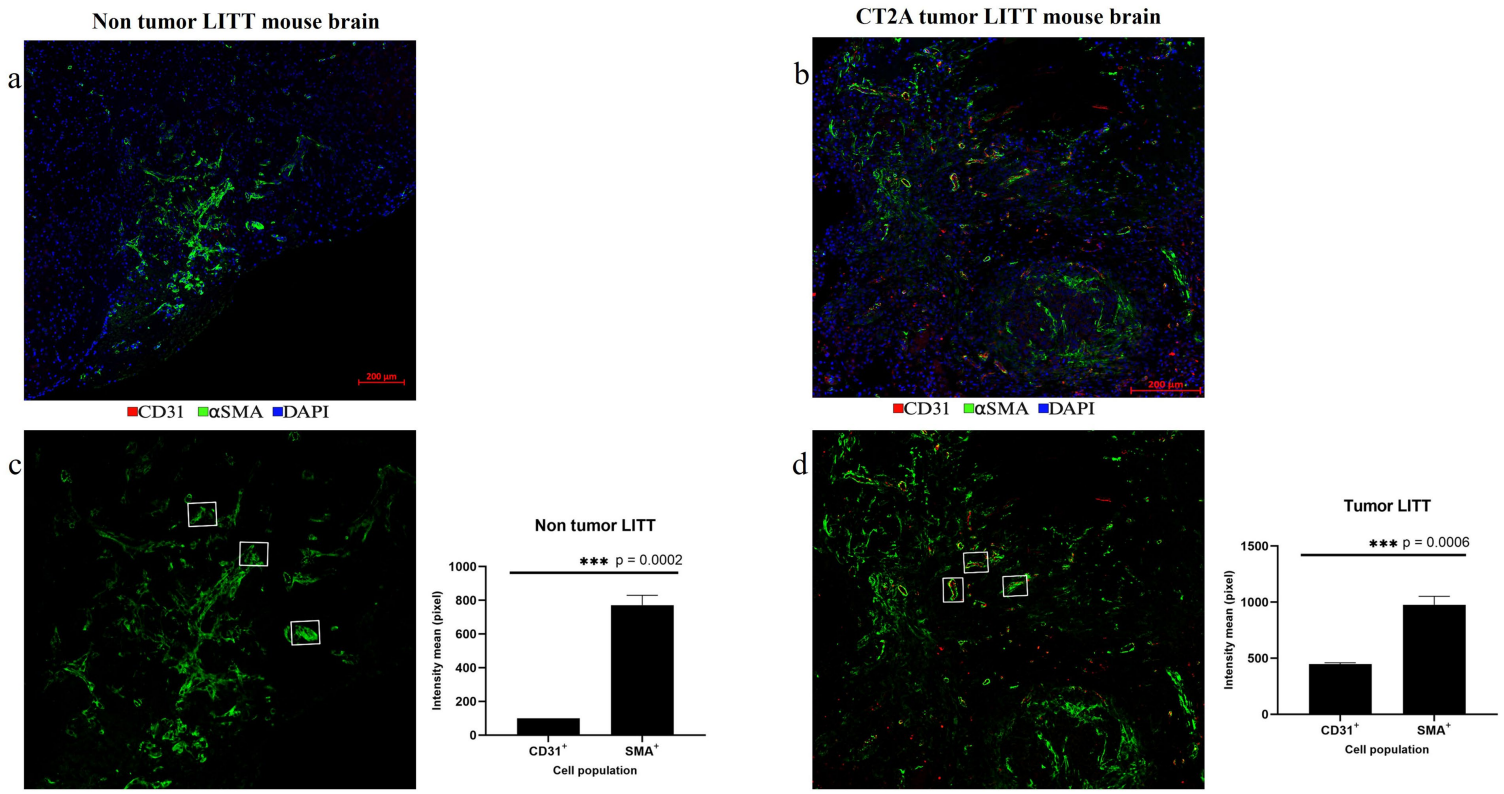
Post-LITT vascular remodeling. **(a)** The upregulation of smooth muscle actin (αSMA; green) in the LITT-treated non-tumor brain suggests post-LITT tissue remodeling. **(b)** Presence of αSma (green) and CD31 (red) was observed in highly vascularized post-LITT CT2A tumor mouse brain. CD31 and ⍺SMA fluorescence intensity mean values were calculated for both **(c)** non-tumor LITT and **(d)** tumor-LITT mouse brain. αSMA upregulation was observed in both models but CD31^+^ endothelial cells were detected exclusively in tumor-LITT sections. Intensity profiles were quantified by selecting 3 αSMA^+^ structures of the same area size from the LITT treated regions of non-tumor and tumor brains. Significance was determined using paired t-test (*n* = 3).

Following LITT probe insertion and treatment-induced damage, tissue repair mechanisms coincided with statistically significant upregulation of αSma in post-LITT normal mouse brains (Figures 12a,c) and highly vascularized CT2A tumor LITT brains (Figures 12b,d). CD31 and αSMA were imaged from two different rounds of staining. The co-localization of these two markers depicts vascular cells and confirms the precision of our alignment technique.

### 3.6 Limitations of multiplex IF on LITT tissue sections

Clinical LITT surgical devices enable integrated real-time temperature measurements during LITT, and live-MR imaging is used for targeted thermal tissue ablation. Current LITT mouse models lack these real-time monitoring features, and the small mouse brain anatomy requires precise surgical coordinates for laser probe insertion into brain tumor tissue. Also, LITT parameters must be optimized to spatially restrict thermal ablation and avoid unintended heat damage to neighboring brain regions.

The limitation of mIF is the number of colors to be used in a single mIF image. It is easy to interpret images with non-overlapping fluorescent colors. When more than two antibodies are used to stain the same cell population the multiplex produces a white color. However, the output channels can be divided into separate multiplex image panels to better illustrate the results. We managed to use the stripping method to achieve an 8-color multiplex image in less than four days. However, assigning appropriate pseudo-colors to elevate each stain can be challenging. Optimal mIF results require strategic planning of the number and sequence of different well-characterized antibodies to be applied and the choice of selected antigenic targets for simultaneous detection in tissues. For example, mIF on a LITT tissue section can be divided into pro-inflammatory and anti-inflammatory multiplex panels that are tested on the same tissue section. The results from a total of 3–4 cycles of immunostaining are then displayed effectively as images of various targeted antigen combinations. This way, the relative spatial distribution of different immune cell populations can be shown in the same LITT brain tissue section. However, LITT-induced heat damage to cells and proteins with central tissue necrosis also poses challenges. This includes false negative (heat-damaged antigens) or false positive (non-specific antibody staining) immunostaining with increased autofluorescence in some LITT tissue regions.

## 4 Concluding remarks

We present a versatile, economical, customizable, and highly reproducible mIF protocol that utilizes a common fluorescence microscopic setup combined with image analysis software to generate high-quality multiplexed fluorescence image datasets with up to 8 well-characterized antibodies targeting selected antigens on a single tissue section. Using our established LITT mouse model (Spence et al., 2024), we selected brain tissues at pre-determined time points post-LITT to demonstrate early cellular responses at the LITT injury site in the mouse brain. This allowed the simultaneous spatial visualization of multiple cellular and molecular targets in a single tissue section and identified region-specific dynamic changes in the composition and activation state of innate phagocytic processes post-LITT at a single cell level. In summary, the combination of mIF and mouse LITT models is a powerful method to study spatial cellular injury-repair processes in the brain.

## Data Availability

The original contributions presented in the study are included in the article/Supplementary material, further inquiries can be directed to the corresponding author.
