# Supplementary figures and images for: A customized affordable multiplexed immunofluorescence method visualizes early changes in the mouse brain microenvironment upon laser cytoreduction
Source: Front Cell Neurosci. 2025 Sep 8;19:1553058. doi: 10.3389/fncel.2025.1553058 (PMC12452037; doi:10.3389/fncel.2025.1553058)

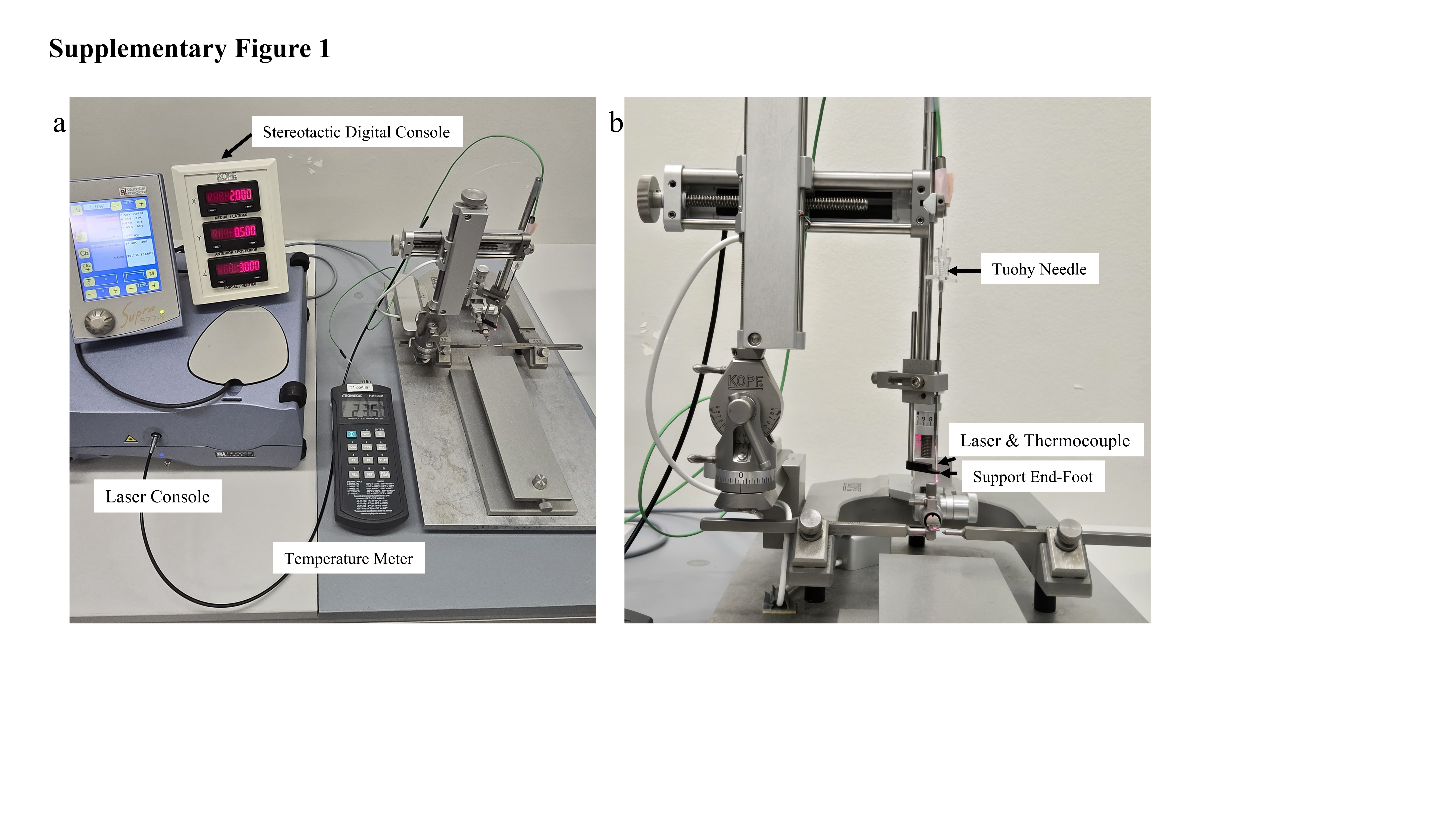

Supplement: Supplementary file 1 [file Image_1.tif]

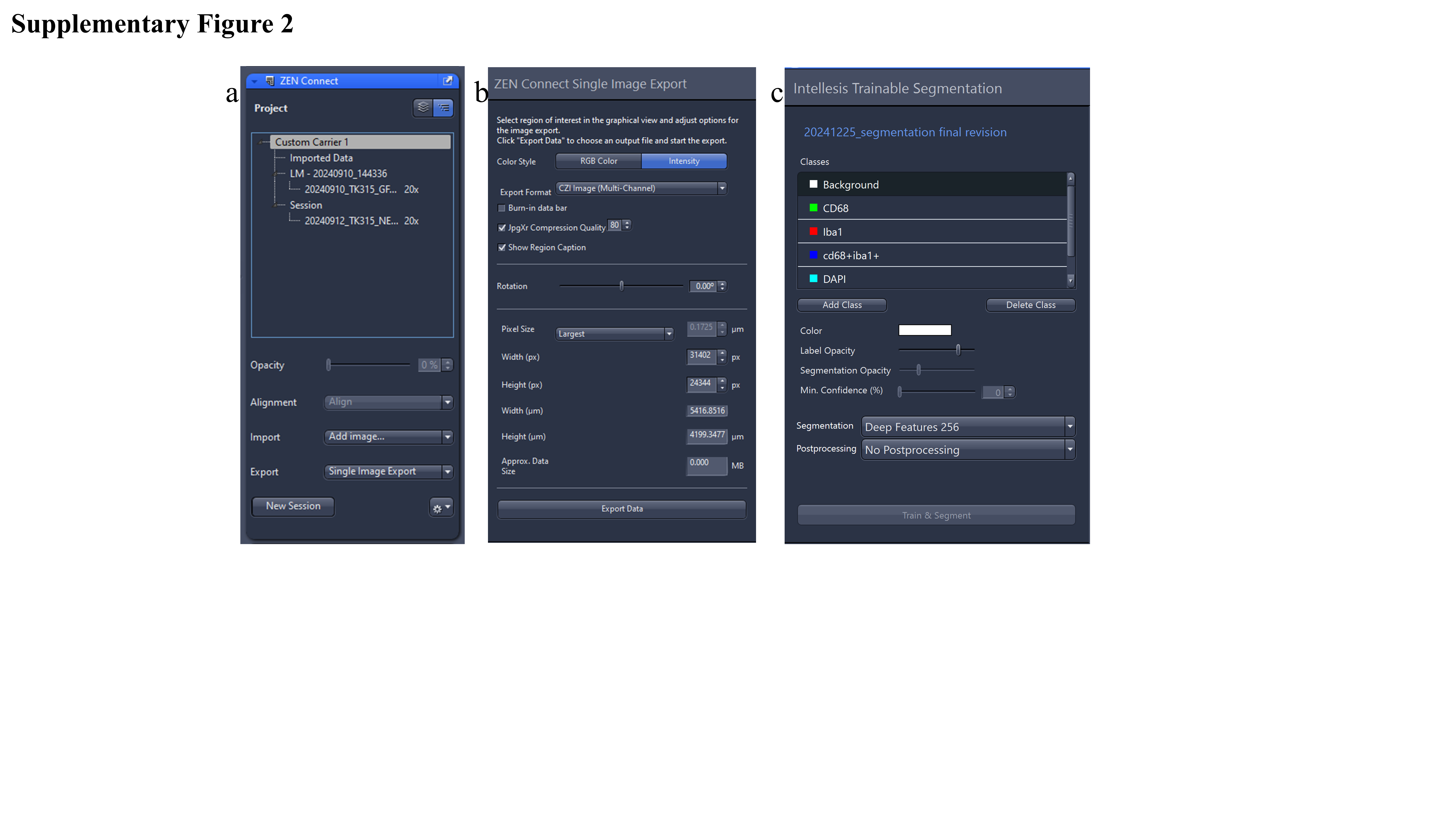

Supplement: Supplementary file 2 [file Image_2.tif]

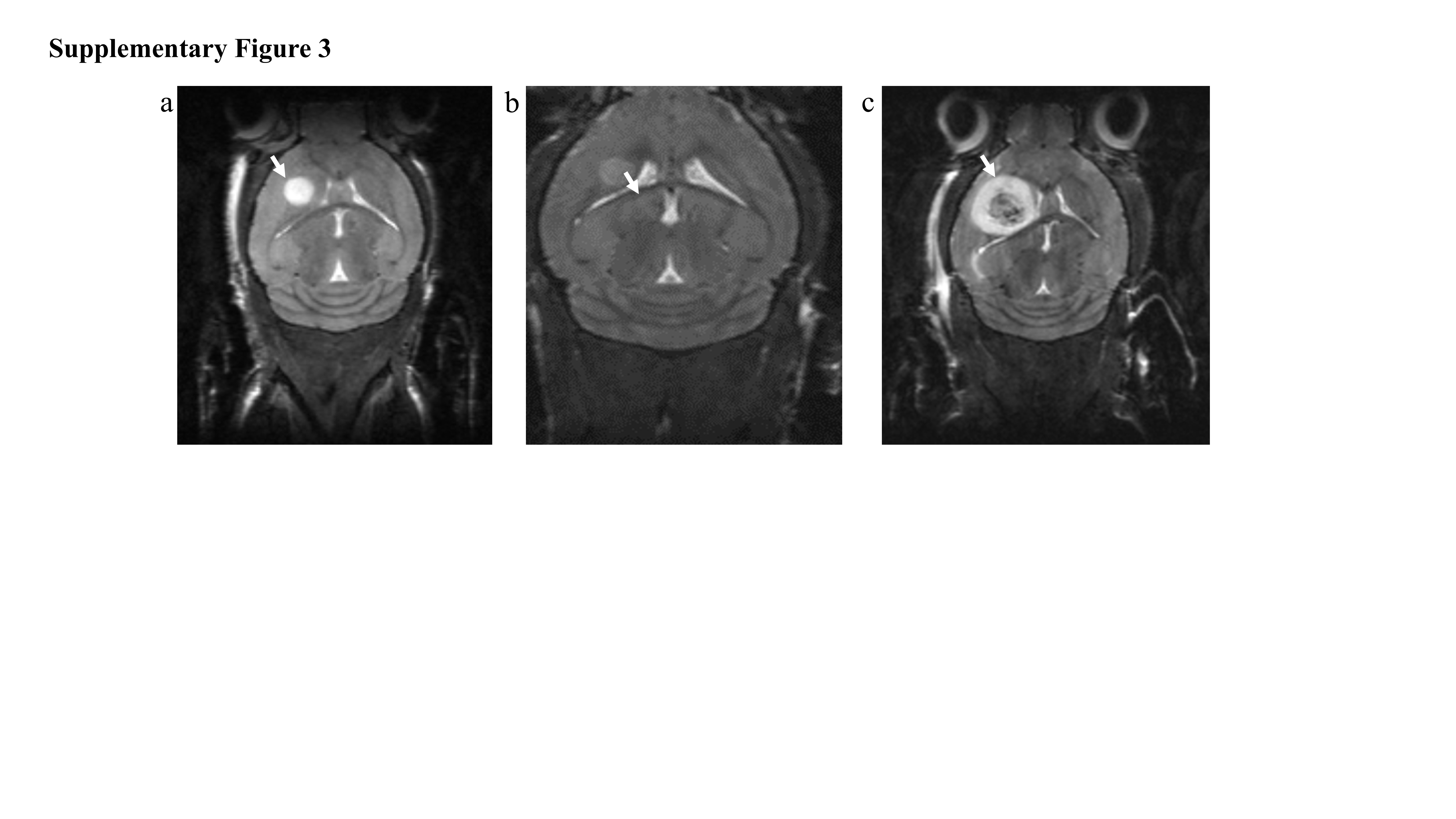

Supplement: Supplementary file 3 [file Image_3.tif]
